# Dominant role of splenic marginal zone lipid rafts in the classical complement pathway against *S. pneumoniae*

**DOI:** 10.1038/s41420-019-0213-3

**Published:** 2019-09-09

**Authors:** Seung Woo Yang, Jin-Yeon Park, Hyeongjwa Choi, Tae Jin Yun, Woo-Sung Choi, Min-Kyung Kim, Yun Kyung Lee, Min Park, Yihwa Jin, Jin Soo Joo, In-Soo Choi, Seung Hwa Park, Han Sung Hwang, Young-Sun Kang

**Affiliations:** 1 Department of Biomedical Science and Technology, Konkuk University, 1 Hwayang-dong, Gwangjin-gu, Seoul 143-701 Republic of Korea; 2 Department of Obstetrics and Gynecology, Division of Maternal and Fetal Medicine, Research Institute of Medical Science, Konkuk University School of Medicine, 120 Neungdong-ro, Gwangjin-gu, Seoul 05029 Korea; 3 Department of Anatomy, Research Institute of Medical Science, Konkuk University School of Medicine, 120 Neungdong-ro, Gwangjin-gu, Seoul 05029 Korea; 4 Department of Oncology, Georgetown University Medical Center, Washington, DC USA; 5 Department of Pathology, New York University School of Medicine, New York, NY 10016 USA; 6 Laboratory Animal Center, KBIO Health, Osongsarngmyon-ro 123, Ghungju-si, Chungbuk Korea; 7 Department of Veterinary Pharmacology and Toxicology, Veterinary Science Research Institute, College of Veterinary Medicine, Konkuk University, 120 Neungdong-ro, Gwangjin-gu, Seoul 05029 Korea; 8 Department of Infectious Diseases, College of Veterinary Medicine, Konkuk University, 120 Neungdong-ro, Gwangjin-gu, Seoul 05029 Korea

**Keywords:** Innate immunity, Complement cascade

## Abstract

Lipid rafts (LRs) play crucial roles in complex physiological processes, modulating innate and acquired immune responses to pathogens. The transmembrane C-type lectins human dendritic cell-specific intercellular adhesion molecule-3-grabbing nonintegrin (DC-SIGN) and its mouse homolog SIGN-R1 are distributed in LRs and expressed on splenic marginal zone (MZ) macrophages. The DC-SIGN-C1q or SIGN-R1-C1q complex could mediate the immunoglobulin (Ig)-independent classical complement pathway against *Streptococcus pneumoniae*. Precise roles of LRs during this complement pathway are unknown. Here we show that LRs are indispensable for accelerating the DC-SIGN- or SIGN-R1-mediated classical complement pathway against *S. pneumoniae*, thus facilitating rapid clearance of the pathogen. The trimolecular complex of SIGN-R1-C1q-C4 was exclusively enriched in LRs of splenic MZ macrophages and their localization was essential for activating C3 catabolism and enhancing pneumococcal clearance, which were abolished in SIGN-R1-knockout mice. However, DC-SIGN replacement on splenic MZ macrophage’s LRs of SIGN-R1-depleted mice reversed these defects. Disruption of LRs dramatically reduced pneumococcal uptake and decomposition. Additionally, DC- SIGN, C1q, C4, and C3 were obviously distributed in splenic LRs of cadavers. Therefore, LRs on splenic SIGN-R1^+^ or DC-SIGN^+^ macrophages could provide spatially confined and optimal bidirectional platforms, not only for usual intracellular events, for example recognition and phagocytosis of pathogens, but also an unusual extracellular event such as the complement system. These findings improve our understanding of the orchestrated roles of the spleen, unraveling a new innate immune system initiated from splenic MZ LRs, and yielding answers to several long-standing problems, including the need to understand the profound role of LRs in innate immunity, the need to identify how such a small portion of splenic SIGN-R1^+^ macrophages (<0.05% of splenic macrophages) effectively resist *S. pneumoniae*, and the need to understand how LRs can promote the protective function of DC-SIGN against *S. pneumoniae* in the human spleen.

## Introduction

Lipid rafts (LRs) are small (10–200 nM), highly dynamic, detergent-resistant membrane fractions enriched in cholesterol and glycosphingolipid content on the plasma membranes of eukaryotic cells^1,2^. Although LRs comprise only a small percentage of the cell surface area^3^, their sizes can increase by coalescence with other raft units^4,5^, providing spatiotemporal platforms for many molecular entities^6^. LRs play crucial roles in complex physiological processes, such as phagocytosis, receptor–receptor associations, receptor–pathogen associations, and signal transduction in many pathological situations^1,2,7–9^, modulating innate and acquired immune responses^10^.

The complement system is important for several innate and adaptive resistance mechanisms and consists of a highly regulated cascade of more than 30 serum complement proteins that can be triggered by the recognition of a microbe^11^. This system can be activated through classical, soluble mannose-binding lectin (MBL), and alternative pathways^12,13^. A pivotal step in the complement pathways is assembly of a C3 convertase, which digests C3 to form microbial binding C3 fragments Because C3 fragments^14^, such as C3b, C3bi, and C3d, serve as ligands for complement receptors, their reciprocal binding promotes the uptake and killing of microbes by phagocytes^15^. Thus, the complement system provides a major extracellular defense mechanism against infectious organisms^16^

Various immune receptors in LRs, such as Fc receptors^17,18^, cytokine receptors^19–21^, B cell receptors^22^, and T cell receptors^23^, increase their binding capacity through clustering^24^ and facilitate signaling to favor the clearance of intracellular pathogens^10^. Some innate pattern recognition receptors, such as Toll-like receptors and transmembrane C-type lectins, translocate to LRs upon stimulation with specific agonists^25–30^, thus demonstrating the importance of this membrane partitioning for the innate immune recognition of various pathogens^3^. In addition, various complement receptors (CR2, CR3, and globular C1q receptor (gC1qR))^31,32^ and complement regulatory proteins (CD46, CD55, and CD59)^33,34^ are distributed in the LRs of immune cells.

Transmembrane C-type lectin human dendritic cell-specific intercellular adhesion molecule-3-grabbing nonintegrin (DC-SIGN, CD209) and its murine homolog SIGN-R1 exhibit several common specificities, such as acting as the principal receptors for the pneumococcal capsular polysaccharide of *S. pneumoniae* (CPS)^35,36^ and human immunodeficiency virus-1^37,38^, binding to the complement C1q^24,39^, and showing distribution in LRs in vitro^29,30^. Additionally, SIGN-R1 can initiate an immunoglobulin (Ig)-independent classical complement pathway by interacting with C1q against *S. pneumoniae*, particularly on the cellular surface of splenic marginal zone (MZ) macrophages, facilitating their rapid clearance^39^. Similarly, the DC-SIGN-C1q complex may provide an initiation site for the classical complement pathway under pathogenic conditions^24^.

Although disruption of LRs significantly reduces DC-SIGN- and SIGN-R1-mediated pneumococcal phagocytosis, the specific mechanisms are still unknown. Accordingly, in this study, the role of LRs was examined in C-type lectin-mediated phagocytosis in order to elucidate the role of DC-SIGN in the human spleen.

## Results

### LRs on splenic MZ DC-SIGN^+^ macrophages may be important for DC-SIGN-mediated uptake and decomposition of *S. pneumoniae*

**Fig. 1:**
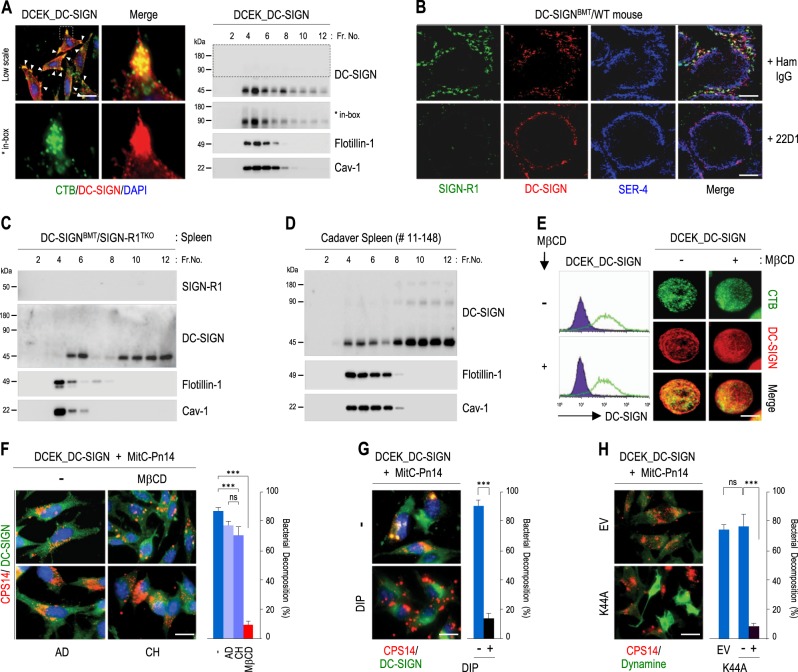
Distribution of DC-SIGN in splenic lipid rafts and its role in the uptake and decomposition of *S. pneumoniae*. **a** (Left) DCEK_DC-SIGN transfectants were immunostained for DC-SIGN (red), cholera toxin B (green), and DAPI (blue). Arrowheads (yellow) indicate the colocalization of DC-SIGN and cholera toxin B. (Right) After sucrose gradient ultracentrifugation of whole-cell lysates from DCEK_DC-SIGN transfectants, fractions were immunoblotted for DC-SIGN, flotilin-1, and caveolin-1. **b** DC-SIGN^BMT^/WT mice were intravenously injected with hamster IgG or 22D1 anti-SIGN-R1 monoclonal antibodies (100 μg, 24 h), and splenic cryosections were immunostained for SIGN-R1 (green), DC-SIGN (red), and SER4/CD169 (blue). **c** As in (**a**) (right), but spleens from DC-SIGN^BMT^/SIGN-R1^TKO^ mice were used, and immunoblotting for SIGN-R1 was performed. **d** As in **a** (right), but cadaver spleens were used, and representative results (#11–148) are presented. **e** (Left) DCEK_DC-SIGN transfectants were treated with MβCD (10 mM, 3 h), immunostained for DC-SIGN without permeabilization, and assessed by FACS. (Right) As in (left), but cells were immunostained for cholera toxin B (green) and DC-SIGN (red), followed by microscopic analysis. **f**–**h** (Left) DCEK_DC-SIGN transfectants were pretreated with **f** MβCD, actinomycin-D (AD; 5 μg/mL, 24 h), or cycloheximide (CH; 20 μg/mL, 24 h) or with **g** the myristoylated dynamin inhibitory peptide (50 μM, 1 h)^70^ and washed out, or **h** transfected with empty vector or dominant-negative dynamin (K44A). Samples were then incubated with mitomycin C-treated *S. pneumoniae* type 14 (MitC-Pn14; 1 × 10^6^, 15 h, 37 °C), followed by immunostaining for **f**, **g** DC-SIGN (green) and CPS14 (red) or **h** dynamin (green) and CPS14 (red). (Right) The average percentage of pneumococcal decomposition of total pneumococcal binding on DC-SIGN transfectants was calculated in five areas from each sample in five independent experiments. Data are shown as mean ± SD. *n.s*., Not significant; *p < 0.05; **p < 0.01; ***p < 0.001. Scale bars **a**, **f**, **g**, **h**, 20 µm; **b**, 200 µm; **e**, 10 µm

DC-SIGN transfectants were immunostained for DC-SIGN and the raft protein GM-1 ganglioside with fluorescein isothiocyanate (FITC)/cholera toxin B (CTB) subunit. Large aggregates of DC-SIGN were strongly distributed in CTB-enriched vertex regions of the cells (Fig. 1a). Moreover, DC-SIGN monomers and dimers were obviously enriched in LR fractions (fractions 4–6) compared with non-LR fractions (Fig. 1a). Isolation of detergent-resistant raft fractions was confirmed with immunoblotting for flotillin-1 and caveolin-1 as representative markers of planar LRs and caveolae, respectively^40^. The same experiment was performed with SIGN-R1 depletion and DC-SIGN transgenic mice (DC-SIGN^BMT^/SIGN-R1^TKO^; Fig. 1b and Supplementary Fig. S1a, b) or human cadavers (Supplementary Fig. S1c). DC-SIGN was obviously expressed in LRs of splenic MZ DC-SIGN^+^ cells (Fig. 1c, d and Supplementary Fig. S1d).

Disruption of LRs with methyl-β-cyclodextrin (MβCD), a cholesterol-extracting agent, did not reduce DC-SIGN expression on the cellular surface, but altered its surface distribution pattern (Fig. 1e). Moreover, when DC-SIGN transfectants were incubated with carboxyfluorescein succinimidyl ester (CFSE)-labeled *S. pneumoniae*, pneumococcal uptake and decomposition were obvious in the cytoplasm, showing disintegrated particles of the pneumococcal capsular polysaccharide of *S. pneumoniae* serotype 14 (CPS14) around the phagocytosed bacterium (Fig. 1f and Supplementary Fig. S1e). Similar results were observed after pretreatment with actinomycin-D or cycloheximide, which did not affect the plasma membrane structure (Fig. 1f). However, pneumococcal uptake and decomposition were dramatically reduced with disruption of LRs using MβCD (Fig. 1f and Supplementary Fig. S1f) or with inhibition of LR-dependent endocytosis using dynamin inhibitory peptide (DIP) or transfection with dominant-negative dynamin (K44A; Fig. 1g and h, respectively), only permitting microbial binding to the cellular surface of DC-SIGN transfectants. The bacterial decomposition ratios were quantitatively calculated (Fig. 1f–h).

### LRs on splenic MZ SIGN-R1^+^ macrophages may be important for SIGN-R1-mediated uptake and decomposition of *S. pneumoniae*

**Fig. 2:**
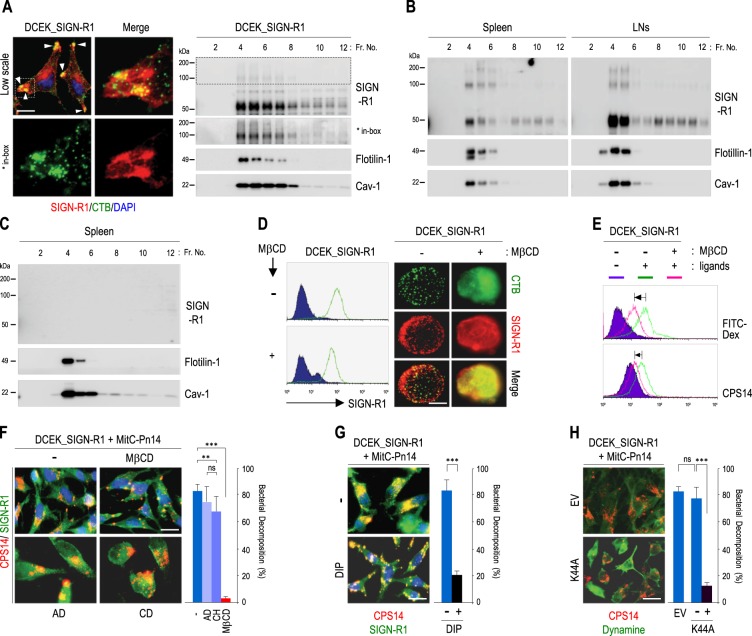
Distribution of SIGN-R1 in splenic lipid rafts and its role in the uptake and decomposition of *S. pneumoniae*. **a** (Left) DCEK_SIGN-R1 transfectants were immunostained for SIGN-R1 (red), cholera toxin **b** (green), and DAPI (blue). Arrowheads (yellow) indicate the colocalization of DC-SIGN and cholera toxin B. (Right) After sucrose gradient ultracentrifugation of whole cell lysates of DCEK_SIGN-R1 transfectants, fractions were immunoblotted for DC-SIGN, flotilin-1, or caveolin-1. Multimers of SIGN-R1 are presented in the box. **b**, **c** As in **a** (right), but spleens from wild-type and SIGN-R1-KO mice were used and immunoblotted for SIGN-R1, flotilin-1, and caveolin-1. **d** (Left) DCEK_SIGN-R1 transfectants were treated with MβCD (10 mM, 3 h), immunostained for SIGN-R1 without permeabilization, and assessed by FACS. (Right) As in (left), but cells were immunostained for cholera toxin B (green) and SIGN-R1 (red) and followed by microscopic analysis. **e** Cells in **d** were further incubated with FITC-dextran (5 μg, 30 min) or CPS14 (10 μg, 2 h), and their uptake was assessed by FACS (green or pink lines for uptake without or with MβCD, respectively). **f**–**h** (Left) DCEK_SIGN-R1 transfectants were pretreated with **f** MβCD, actinomycin-D (AD; 5 μg/mL, 24 h), cycloheximide (CD; 20 μg/mL, 24 h) or with **g** myristoylated dynamin inhibitory peptide (50 μM, 1 h)^71^ and washed out, or **h** transfected with dominant-negative dynamin (K44A) and incubated with mitomycin C-treated *S. pneumoniae* type 14 (MitC-Pn14; 1 × 10^6^, 15 h, 37 °C), followed by immunostaining for **f**, **g** SIGN-R1 (green) and CPS14 (red) or (H) dynamin (green) and CPS14 (red). (Right) The average percentage of pneumococcal decomposition of total pneumococcal binding on SIGN-R1 transfectants was calculated in five areas from each sample in five independent experiments. Data are shown as mean ± SD. n.s., Not significant; *p < 0.05; **p < 0.01; ***p < 0.001., Scale bars **a**, **f**, **g**, **h**, 20 µm; **d**, 10 µm

Large aggregates of SIGN-R1 were observed in CTB-enriched vertex regions of SIGN-R1 transfectants and strong distribution of SIGN-R1 monomers and dimers were obvious in LR fractions (Fig. 2a). In particular, multimers of SIGN-R1 were preformed in LRs (inset in Fig. 2a). In whole fractions of spleens or lymph nodes from wild-type (WT) mice, SIGN-R1 was evident only in LRs of both tissues of WT mice with a higher concentration of SIGN-R1 multimers in LRs than in non-LRs (Fig. 2b), but not from SIGN-R1-knockout (KO) mice (Fig. 2c and Supplementary Fig. S2a).

Disruption of LRs with MβCD did not reduce SIGN-R1 expression on the transfectant cellular surface, but altered its surface distribution pattern (Fig. 2d). However, MβCD treatment of SIGN-R1 transfectants reduced the uptake of dextran or CPS14, representative ligands of SIGN-R1^41,42^ (Fig. 2e). Similarly, when SIGN-R1 transfectants were incubated with *S. pneumonia* type 14, which has strong binding affinity for SIGN-R1^41^, the uptake and decomposition of the organism were evident under control conditions (Fig. 2f and Supplementary Fig. S2b) and in the presence of actinomycin-D or cycloheximide (Fig. 2f). However, MβCD treatment of SIGN-R1 transfectants inhibited the uptake and decomposition of *S. pneumoniae*, only permitting microbial binding to the cellular surface (Fig. 2f). Moreover, inhibition of LR-dependent endocytosis using DIP or K44A dramatically reduced the uptake and decomposition of the organism (Fig. 2g, h). The bacterial decomposition ratios were quantitatively calculated (Fig. 2f–h).

### LRs of splenic MZ SIGN-R1^+^ macrophages may provide an optimal location for innate recruitment of SIGN-R1 against *S. pneumoniae* in vivo

**Fig. 3:**
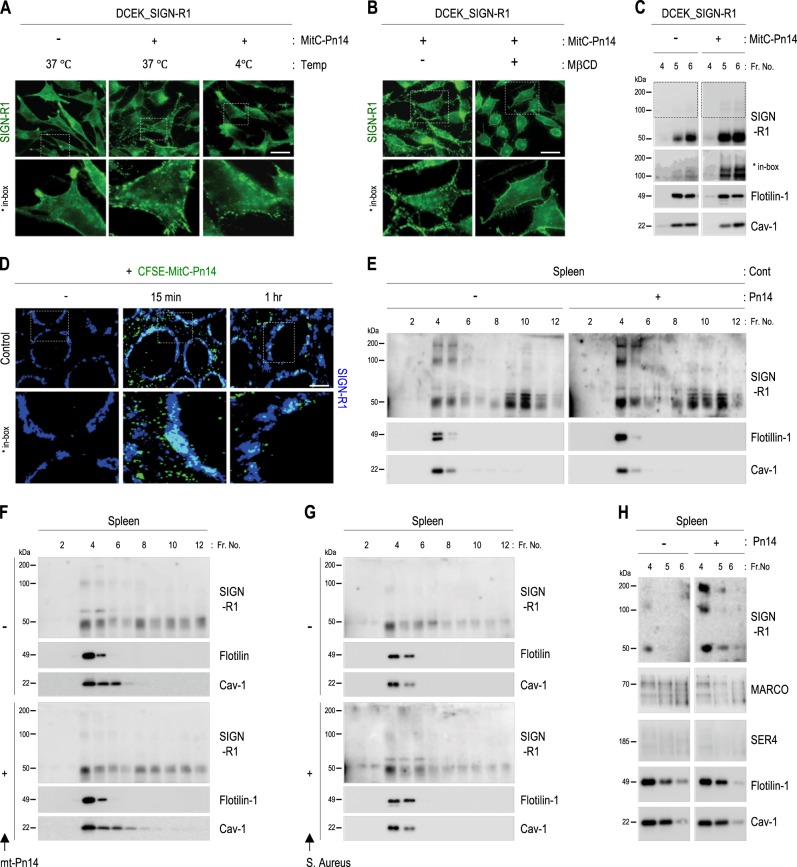
Accumulation and multimerization of SIGN-R1 in splenic lipid rafts following exposure to CPS14 from *S. pneumoniae* in vivo. **a** DCEK_SIGN-R1 transfectants were incubated with mitomycin C-treated *S. pneumoniae* type 14 (MitC-Pn14; 1 × 10^6^, 10 min) at 37 °C or 4 °C and immunostained for SIGN-R1 without permeabilization. **b** As in **a**, but cells were pretreated with MβCD (10 mM, 3 h) and incubated only at 37 °C. **c** As in **a**, but cell lysates at 37 °C were fractionated with sucrose gradient ultracentrifugation, and fractions of LRs were immunoblotted for SIGN-R1, flotilin-1, or caveolin-1. Multimers of SIGN-R1 are shown in the boxes. **d** In total, 1 × 10^8^ CFSE-labeled MitC-Pn14 (green) were injected intravenously into wild-type mice for 0, 15, or 60 min, and splenic sections were immunostained for SIGN-R1 (blue). The binding or uptake of organisms into splenic MZs is shown in the boxes. (E) As in (C), but spleens were used before or after intravenous injection of live *S. pneumoniae* (Pn14; 1 × 10^8^, 1 hr) into wild-type mice. (F and G) As in **e**, but mice were injected intravenously with PBS or 1 × 10^8^ cells of an unencapsulated mutant of serotype 14 *S. pneumoniae* (mt-Pn14) or *Staphylococcus aureus* for 1 h, respectively. **h** As in **e**, but fractions were immunoblotted for MARCO, SER4/CD169, flotilin-1, and caveolin-1. Scale bars **a**, **b**, 20 µm; **d**, 250 µm

SIGN-R1 transfectants were incubated with *S. pneumoniae* at 37 °C or 4 °C or in the presence of MβCD, and abundant SIGN-R1 aggregation was observed on the cell surface only at 37 °C (Fig. 3a, b). When these cells were then fractionated and their LR fractions were immunoblotted for SIGN-R1, SIGN-R1 monomers and multimers were obviously increased in LRs (Fig. 3c). Because SIGN-R1^+^ macrophages rapidly recognized *S. pneumoniae* in splenic MZs within 1 h (Fig. 3d), SIGN-R1 distribution in splenic LRs was examined 1 h after intravenous injection of *S. pneumoniae*. SIGN-R1 complex was obviously increased only in splenic LRs following *S. pneumoniae* stimulation (Fig. 3e), as confirmed in separate experiments (Supplementary Fig. S3a, cases 1–4).

Similar experiments using intravenous injection of an unencapsulated mutant of *S. pneumoniae* serotype 14 (mt-*Pn14*) or *Staphylococcus aureus*, another gram-positive coccal bacterium that does not bind SIGN-R1^41,43^ showed no increase in SIGN-R1 complex in splenic LRs (Fig. 3f, g), confirming the CPS14 dependent recruitment of SIGN-R1 complex against *S. pneumoniae*. Next, we examined whether MARCO, a scavenger receptor expressed on partial SIGN-R1^+^ MZ macrophages^44^, or SER4/CD169, a cell adhesion molecule expressed on MZ metallophils, were recruited in splenic LRs following *S. pneumoniae* exposure. Neither target was recruited in splenic LRs following exposure to *S. pneumoniae* (Fig. 3h).

### C1q and C4 are distributed in LRs of splenic MZ SIGN-R1^+^ macrophages and increased following *S. pneumoniae* exposure in a SIGN-R1-dependent manner in vivo

**Fig. 4:**
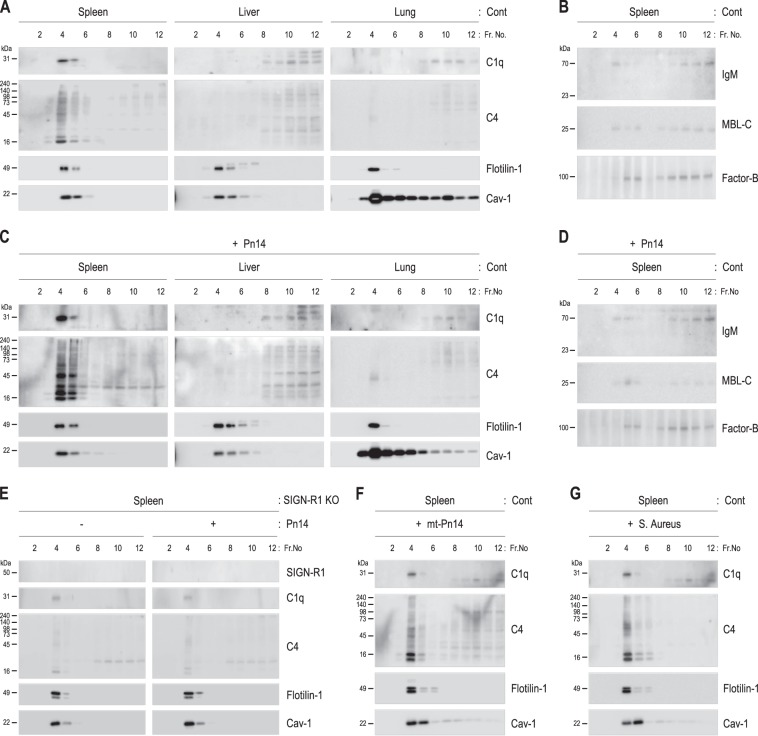
Predominant distribution of complement C1q and C4 in splenic lipid rafts and their upregulation after *S. pneumoniae* challenge in a SIGN-R1-dependent manner in vivo. **a** Spleens, livers, and lungs of control mice were fractionated with sucrose gradient ultracentrifugation, and fractions were immunoblotted for C1q, C4, flotilin-1, and caveolin-1. **b** As in **a**, but fractions were immunoblotted for IgM, MBL-C, or factor B. **c** As in **a**, but wild-type mice were injected intravenously with live *S. pneumoniae* (Pn14; 1 × 10^8^, 1 h). **d** As in **c**, but fractions were immunoblotted for IgM, MBL-C, or factor B. **e** As in **a**, **c**, but SIGN-R1-KO mice were used, and splenic fractions were analyzed. **f**, **g** As in **c**, but mice were injected intravenously with an unencapsulated mutant of serotype 14 *S. pneumoniae* (mt-Pn14) or *Staphylococcus aureus* (1 × 10^8^, 1 h). All data are representative of five independent experiments

The expression levels of C1q and C4 in whole-cell lysates from the spleens, livers, and lungs of control mice were similar in all tissues (Supplementary Fig. S4b). However, higher levels of C1q and C4 were found in splenic LRs than in hepatic or pulmonary LRs or in non-LRs from all tissues (Fig. 4a). Other traditional mediators in different complement pathways (IgM, MBL-C, and factor B) were barely present in LRs from all tissues (Fig. 4b), but were variable in non-LRs (Supplementary Fig. S4c). Following intravenous injection of phosphate-buffered saline (PBS) or *S. pneumoniae*, simultaneous increases in C1q and C4 were obvious only in splenic LRs, whereas no changes were found in hepatic or pulmonary LRs or in non-LRs (Fig. 4c). Additionally, no differences in IgM, MBL-C, or factor B levels were found in any fraction (Fig. 4d and Supplementary Fig. S4d).

In SIGN-R1-KO mice, the levels of C1q and C4 in whole-cell lysates of all tissues were the same as those in control mice (Supplementary Fig. S4e, b). However, both complements were not increased at all following *S. pneumoniae* exposure (Fig. 4e). Moreover, increases in C1q and C4 were completely abolished in splenic LRs from SIGN-R1^TKO^ mice exposed to *S. pneumoniae* (Supplementary Fig. S4f). To determine whether the specific recognition of CPS14 by SIGN-R1 was required for upregulation of C1q and C4 in splenic LRs, splenic fractions were immunoblotted for C1q and C4. Both complements were not upregulated at all in any splenic fraction following exposure to mt-*Pn14* or *S. aureus* (Fig. 4f, g).

### LRs from splenic MZ SIGN-R1^+^ macrophages may provide an optimal location for dominant C3 activation in response to *S. pneumoniae* in a SIGN-R1-dependent manner in vivo

**Fig. 5:**
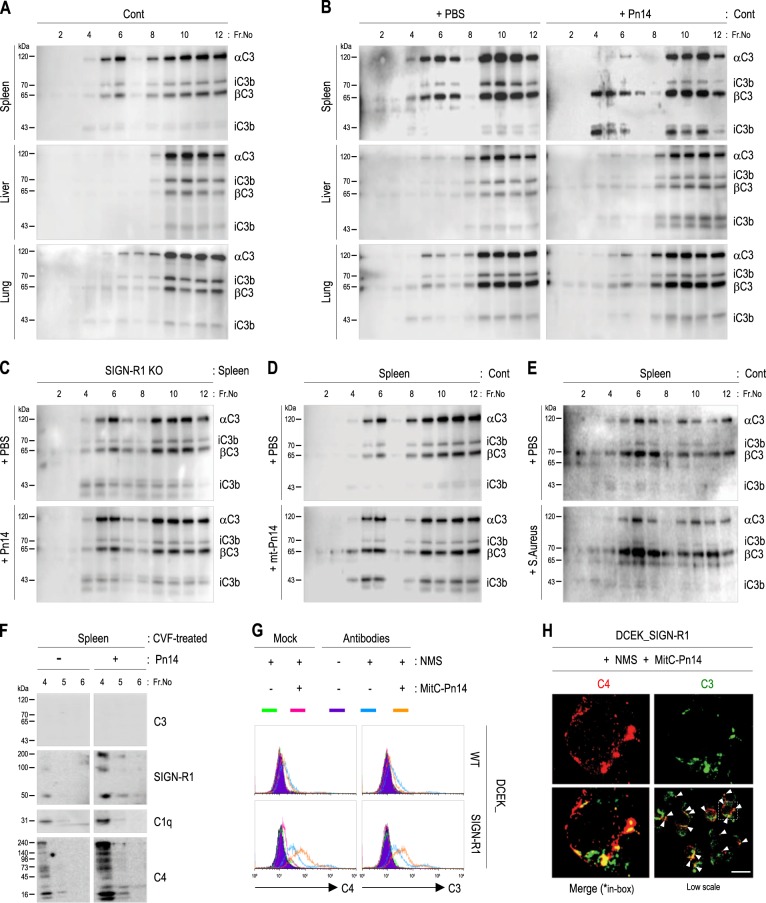
Dominant C3 activation in splenic lipid rafts in a SIGN-R1-dependent manner following *S. pneumoniae* challenge in vivo. **a** Spleens, livers, and lungs of control mice were fractionated with sucrose gradient ultracentrifugation, and fractions were immunoblotted for C3. **b** As in **a**, but wild-type mice were injected intravenously with PBS or live *S. pneumoniae* (Pn14; 1 × 10^8^, 1 h). **c** As in **a**, **b**, but SIGN-R1-KO mice were used, and splenic fractions were analyzed. **d**, **e** As in **c**, but mice were injected intravenously with an unencapsulated mutant of serotype 14 *S. pneumoniae* (mt-Pn14) or *Staphylococcus aureus* (1 × 10^8^, 1 h). **f** As in **c**, but CVF-treated mice were used, and splenic fractions were immunoblotted for C3, SIGN-R1, C1q, and C4. **g** DCEK_WT and DCEK_SIGN-R1 were incubated with mitomycin C-treated *S. pneumoniae* type 14 (MitC-Pn14; 1 × 10^7^, 1 h) without or with 5% NMS in TC buffer for 15 min at 37 °C. Cells were immunostained for C4 or C3 with their respective isotype control IgGs and assessed by FACS. **h** As in **g**, but the binding of C4 (red) or C3 (green) was assessed by fluorescence microscopy after fixing DCEK-SIGN-R1 transfectants with 1% paraformaldehyde. Arrowheads indicate colocalization of C4 and C3. Representative cells are shown in the box. All data are representative of five independent experiments. Scale bars **h**, 10 µm

We examined C3 levels in whole-cell lysates from the spleen, liver, and lung of WT mice, and the lowest expression was observed in the spleen (Supplementary Fig. S5a). However, immunoblotting of their fractions for C3 showed that constitutive distribution of C3 was the highest in splenic LRs among LRs of all tissues (Fig. 5a). Furthermore, following intravenous injection of PBS or *S. pneumoniae*, the initial activation of C3 was most dominant in splenic LRs exposed to *S. pneumoniae* with a dramatic decrease in αC3, but a relatively minor decrease was observed in splenic non-LRs (Fig. 5b). These results in spleen fractions were confirmed in separate experiments (Supplementary S5b, c).

In sera or splenic fractions from SIGN-R1-KO or SIGN-R1^TKO^ mice, SIGN-R1 deficiency did not affect C3 levels in sera (Supplementary S5g) or distribution in splenic LRs without pneumococcal challenge (Fig. 5c, top, Supplementary Fig. S5f, g, top), but abolished C3 activation with pneumococcal challenge (Fig. 5c, bottom and Supplementary Fig. S5g, bottom). Moreover, CPS14 was essential for SIGN-R1-mediated C3 activation in splenic LRs because C3 activation was significantly decreased or abolished in all splenic fractions from WT mice following mt-*Pn14* or *S. aureus* challenge, yielding abundant intact C3α and weak or no increases in 43 kDa iC3b (Fig. 5d, e). In C3-depleted mice, C3 deficiency in splenic LRs had no effect on the constitutive distribution of SIGN-R1, C1q, or C4 in splenic LRs or their increases against *S. pneumoniae* (Fig. 5f).

When WT cells or SIGN-R1 transfectants were incubated with mouse serum, fixation of C4 and C3 was observed only on SIGN-R1 transfectants and was significantly enhanced in SIGN-R1 transfectants in the presence of *S. pneumoniae* (Fig. 5g). Furthermore, fluorescent microscopy revealed the colocalization of C4 and C3 only in SIGN-R1 transfectants (Fig. 5h and Supplementary Fig. S5h), indicating that C3 activation was specifically generated at the same location on SIGN-R1^+^ cells in which the SIGN-R1-mediated classical complement pathway was initiated.

### Enrichment of SIGN-R1 and C1q enhances the opsonization, uptake, and decomposition of *S. pneumoniae*

**Fig. 6:**
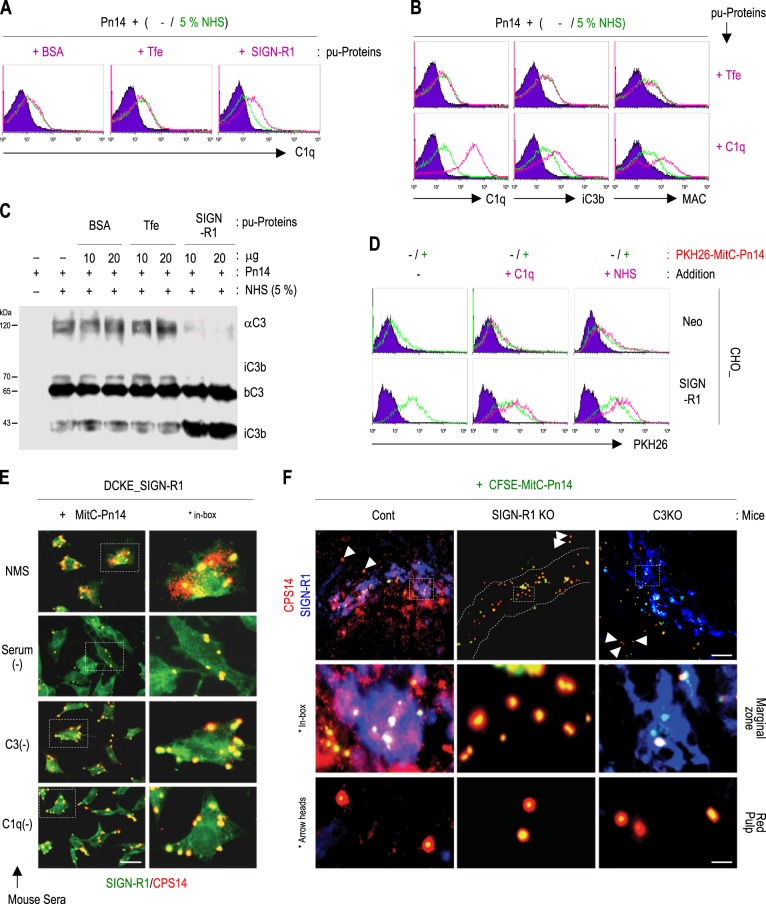
SIGN-R1-mediated C3 activation and opsonization of *S. pneumoniae* enhances the uptake and decomposition of the bacteria by SIGN-R1^+^ cells in vitro and in vivo. **a** Live *S. pneumoniae* type 14 (Pn14) was incubated with 5% NHS at 37 °C for 30 min in the presence of 10 μg bovine serum albumin, transferrin (Tfe), or purified SIGN-R1, immunostained for C1q, and analyzed by FACS. **b** As in **a**, but 10 μg Tfe or C1q was used, and organisms were immunostained for C1q, iC3b, or membrane attack complex (MAC). **c** As in **a**, but organisms were immunoblotted for C3. **d** In total, 1 × 10^8^ mitomycin C-treated and PKH26-labeled *S. pneumoniae* (PKH26-MitC-Pn14) were incubated with CHO transfectants in the presence of 10 μg C1q or 5% NHS. The binding of organisms to cells was assessed by FACS. **e** DCEK_SIGN transfectants were incubated with MitC-Pn14 (1 × 10^6^, 15 h, 37 °C) without or with mouse sera (NMS or C3- or C1q-depleted mouse sera), and cells were immunostained for SIGN-R1 (green) or CPS14 (red), followed by microscopic analysis. Representative cells are shown in the boxes. **f** CFSE-MitC-Pn14 (1 × 10^8^, green) were injected intravenously into control, SIGN-R1-KO, or C3-KO mice for 1 h and spleen sections were immunostained for SIGN-R1 (blue) and CPS14 (red). Representative areas in splenic MZs are highlighted in the boxes (middle row). Organisms captured in the red pulp (arrowheads) are highlighted (bottom row). Scale bars **e**, 20 µm; **f**, 50 µm

Addition of SIGN-R1 significantly enhanced the fixation of C1q on the pneumococcal surface (Fig. 6a). Sequentially, the increased concentration of C1q also accelerated the opsonization of iC3b in response to *S. pneumoniae* and led to the formation of a membrane attack complex on *S. pneumoniae* (Fig. 6b). Because the same amount of βC3 was used for fixation, the same amount of C3 was fixed on *S. pneumoniae* under all conditions. However, C3 activation was dominant only with the addition of SIGN-R1, demonstrating the degradation of αC3 and increased fixation of small iC3b (43 kDa; Fig. 6c).

With the addition of C1q or normal human serum (NHS) as a source of C1q, pneumococcal binding was increased only on SIGN-R1 transfectants (Fig. 6d). Moreover, pneumococcal uptake and sequential decomposition were observed on SIGN-R1 transfectants only with normal mouse serum (NMS), but not without serum or with C3- or C1q-depleted sera (Fig. 6e and Supplementary Fig. S6a). After intravenous injection of *S. pneumoniae* into WT, SIGN-R1-KO, or C3-KO mice, pneumococcal uptake and cytoplasmic decomposition were obvious only on splenic MZ SIGN-R1^+^ macrophages from control mice (Fig. 6f and Supplementary Fig. S6b), but completely abolished in splenic MZs from SIGN-R1-KO and C3-KO mice and red pulp from all mice (Fig. 6f). The signal specificity of CPS14 on splenic MZ SIGN-R1^+^ macrophages of control mice in Fig. 6f was confirmed by immunostaining with its respective isotype control immunoglobulins (Supplementary Fig. S6c).

### DC-SIGN may mediate the classical complement pathway in LRs from splenic MZ DC-SIGN^+^ macrophages following *S. pneumoniae* challenge via interactions with C1q

**Fig. 7:**
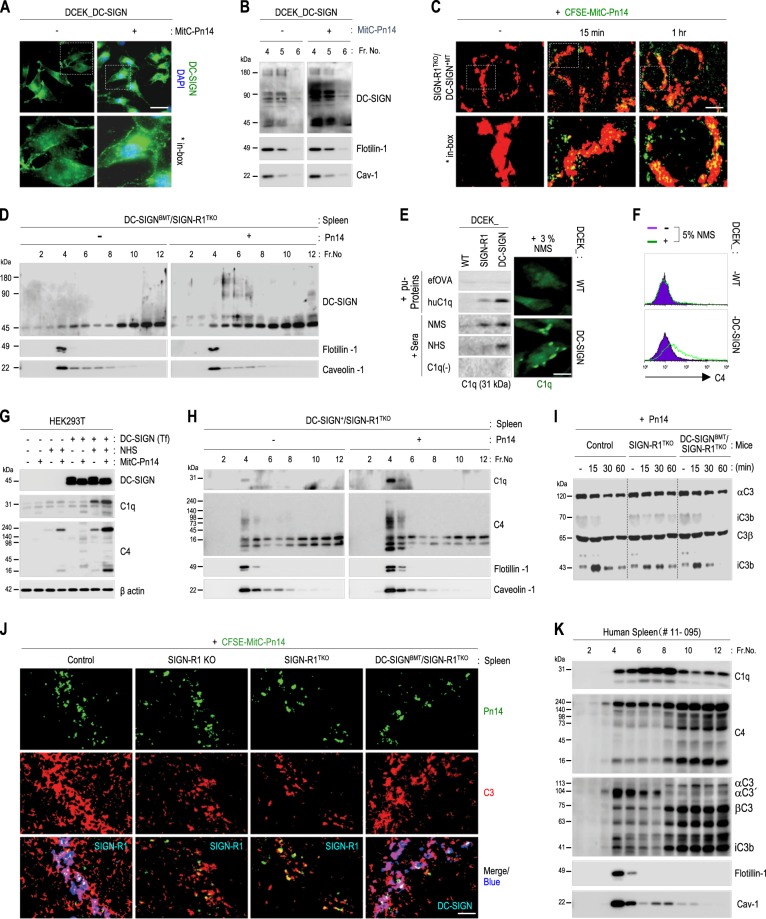
DC-SIGN in splenic lipid rafts may mediate the classical complement pathway in response to *S. pneumoniae* challenge by interacting with C1q and C4. **a** DCEK_DC-SIGN transfectants were incubated with mitomycin C-treated *S. pneumoniae* type 14 (MitC-Pn14; 1 × 10^6^, 15 min, 37 °C) and immunostained for DC-SIGN without permeabilization. A representative cell is shown in the box. **b** After DCEK_DC-SIGN transfectants were incubated with MitC-Pn14 (1 × 10^6^, 1 h, 37 °C), lysates were fractionated with sucrose gradient ultracentrifugation, and fractions of LRs were immunoblotted for DC-SIGN, flotilin-1, or caveolin-1. **c** DC-SIGN^BMT^/SIGN-R1^TKO^ mice were injected intravenously with CFSE-labeled MitC-Pn14 (green; 1 × 10^8^, 15 min or 1 h), and splenic sections were immunostained for DC-SIGN (red). **d** As in **b**, but spleens of DC-SIGN^BMT^/SIGN-R1^TKO^ mice were used after intravenous injection of live *S. pneumoniae* (1 × 10^8^, 1 h). **e** (Left) Wild-type or DCEK transfectants were incubated with 5 μg endotoxin-free ovalbumin (efOVA), human C1q, or 3% sera (NMS, NHS, or C1q-depleted human serum) in TC buffer for 1 h at 37 °C, and lysates were immunoblotted for C1q. (Right) Wild-type or DCEK_DC-SIGN transfectants were incubated with 3% NMS for 1 h at 37 °C and immunostained for C1q, followed by microscopic analysis. Representative areas are highlighted from low-power images in Figure S7a. **f** Wild-type or DCEK transfectants were incubated with 5% NMS for 10 min at 37 °C, immunostained for C4 without permeabilization, and analyzed by FACS. **g** Empty vector or DC-SIGN-transfected HEK293T cells were incubated without or with MitC-Pn14 (1 × 10^7^) in the absence or presence of 5% NHS for 15 min at 37 °C, and cell lysates were immunoblotted for DC-SIGN, C1q, C4, and β-actin. **h** As in **d**, but DC-SIGN^BMT^/SIGN-R1^TKO^ mice were used, and splenic fractions were immunoblotted for C1q or C4. **i** Control, SIGN-R1^TKO^, and DC-SIGN^BMT^/SIGN-R1^TKO^ mice were injected intravenously with Pn14 (1 × 10^8^), and their sera were collected at 0, 15, 30, or 60 min and immunoblotted for C3. **j** As in **c**, but control, SIGN-R1 KO, SIGN-R1^TKO^, and DC-SIGN^BMT^/SIGN-R1^TKO^ mice were intravenously injected for 15 min. Splenic sections were immunostained for C3 (red) and SIGN-R1 (green) or C3 (red) and DC-SIGN (green). Representative areas are highlighted from low-power images in Figure S7b. **k** As in **h**, but spleens of cadavers were used and immunoblotted for C1q, C4, and C3. Representative data are presented from the results of four cadavers. Scale bars **a**, **e**, 20 µm; **c**, 250 µm; **j**, 50 µm

When DC-SIGN transfectants were treated with *S. pneumoniae*, DC-SIGN aggregates were dramatically increased on the cell surface (Fig. 7a). Additionally, when LR fractions of these cells were immunoblotted for DC-SIGN, monomers and dimers of DC-SIGN were obviously increased in LRs following *S. pneumoniae* challenge (Fig. 7b). Moreover, splenic MZ DC-SIGN^+^ macrophages from DC-SIGN^BMT^/SIGN-R1^TKO^ mice recovered the rapid recognition of *S. pneumoniae* on splenic MZs, even at 15 min and 1 h after intravenous injection of the organisms (Fig. 7c). In spleens at 1 h, DC-SIGN monomers and multimers were obviously upregulated only in splenic LRs following *S. pneumoniae* challenge (Fig. 7d).

DC-SIGN bound well to purified human C1q or human C1q from NHS (Fig. 7e). Additionally, DC-SIGN binding to mouse C1q from NMS was confirmed by immunoblotting and immunostaining (Fig. 7e and Supplementary Fig. S7a). We then examined whether DC-SIGN bound directly to C4, similar to SIGN-R1^39^ (Supplementary Fig. S4a). DC-SIGN transfectants showed clear fixation of C4 (Fig. 7f). Additionally, DC-SIGN-transfected HEK293T cells also induced the fixation of C1q or C4 from NHS, confirming again the binding of DC-SIGN to C1q and C4 (Fig. 7g), and their fixation was significantly increased following *S. pneumoniae* challenge (Fig. 7g).

In splenic fractions from DC-SIGN^BMT^/SIGN-R1^TKO^ mice, upregulation of C1q and C4 was obvious following *S. pneumoniae* challenge (Fig. 7h). Moreover, after intravenous injection of *S. pneumoniae* into DC-SIGN^BMT^/SIGN-R1^TKO^ mice, systemic C3 activation reappeared in SIGN-R1^TKO^ mice as in control mice, demonstrating gradual decrease in αC3 and larger iC3b and a rapid increase and sequential decrease in small iC3b (Fig. 7i). C3 fixation was also completely recovered around splenic MZ DC-SIGN^+^ macrophages in DC-SIGN^BMT^/SIGN-R1^TKO^ mice from SIGN-R1-KO or SIGN-R1^TKO^ mice as in control mice (Fig. 7j and Supplementary Fig. S7b), occurring rapidly within 15 min after challenge with *S. pneumoniae* and disappearing at 4 h (Supplementary Fig. S7c). Immunostaining with isotype control IgG for anti-C3 antibodies did not yield a C3 signal (Supplementary Fig. S7d). Moreover, immunoblotting of cadaver spleens for C1q, C4, and C3 revealed expression in human splenic LRs, with predominant expression of C1q and 104-kDa αC3′ rather than 113-kDa αC3 (Fig. 7k and Supplementary Fig. S7e).

## Discussion

Although DC-SIGN and SIGN-R1 professionally recognize and remove *S. pneumonia*^35,41^, the disruption of LRs on both lectin transfectants resulted in serious impairment of the uptake and sequential decomposition of the organism. Because the disruption of LRs did not alter the expression levels of both lectins on the respective transfectants, which would lead to normal binding of the organism to the cell surface, the structure of LRs may be more important for the function of both lectins and for removal of *S. pneumoniae* than previously thought. This speculation was strongly supported by the following results. First, multimers of DC-SIGN or SIGN-R1 were preformed in LRs of the respective transfectants, and even SIGN-R1 multimers were preformed in splenic LRs. Additionally, DC-SIGN and SIGN-R1 multimers were increased only in LRs following *S. pneumoniae* challenge in vitro or in vivo, but not in non-LRs. Therefore, LRs could provide a spatially confined and optimal platform for DC-SIGN and SIGN-R1 to facilitate clustering, increase their affinity and specificity to pathogens, and enhance their clearance, thus affecting their innate protective functions^7,45^.

Raft locations for target antigens favor classical complement activation by concentrating the antigen–antibody complex into a comparatively small area, thus providing an ideal density for juxtaposed Fc regions to engage C1q^46,47^. Similarly, both C1q and C4 were exclusively distributed in splenic LRs among fractions from examined mouse tissues, showing a significant dependence on the existence of SIGN-R1. SIGN-R1 was exclusively expressed on splenic MZ macrophages^42^ and bound directly to C1q or C4^39,48^. Additionally, there is a potential interaction between C1q and C4^49,50^. Therefore, their exclusive distribution in splenic LRs is expected, and these proteins may be further subdivided into splenic MZ SIGN-R1^+^ LRs from heterogeneous LRs of various splenic cells^51^. These findings suggested that splenic MZ SIGN-R1^+^ LRs may provide optimal microenvironmental platforms for reciprocal interactions among SIGN-R1, C1q, and C4, thus preforming a trimolecular complex to rapidly activate the SIGN-R1-mediated classical complement pathway in the spleen.

Because SIGN-R1 or C1q directly recognize *S. pneumoniae*^41,52^, the preformed trimolecular complex of SIGN-R1, C1q, and C4 in splenic MZ SIGN-R1^+^ LRs could accelerate the generation of a classical pathway C3 convertase (C4bC2a), and the resulting abundant C4b and C4bC2a could sequentially promote fixation of C3 on the cell surface^53–56^. Supporting this possibility, C3, particularly C3α, was most enriched in splenic LRs, despite having lowest expression in the spleen among examined mouse tissues. Therefore, splenic MZ SIGN-R1^+^ LRs may be optimized to activate C3, although the C3 distribution was independent of the existence of SIGN-R1, probably due to covalent fixation of C3 fragments on other splenic cells^57^. Because the complement system should be tightly controlled under physiological conditions to prevent significant damage to self-tissues, enrichment of SIGN-R1, C1q, C4, and C3 in splenic MZ SIGN-R1^+^ LRs could also precisely regulate complement activation in splenic MZs; indeed, nascent C3b and C4 regulate C1 activation in concert with C3 under normal conditions^58^.

Pneumococcal challenge increased both C1q and C4 and activated C3 only in splenic MZ SIGN-R1^+^ LRs without the involvement of other complement pathways. Therefore, splenic MZ SIGN-R1^+^ LRs may provide optimal locations for SIGN-R1-mediated C3 activation to accelerate pneumococcal clearance by sequentially enhancing pneumococcal iC3b opsonization and rapid phagocytosis primarily in splenic MZ SIGN-R1^+^ macrophages. In particular, *S. pneumoniae* capsules inhibit its recognition by natural IgM, the binding of the serum proteins to subcapsular targets, complement activity, and neutrophil phagocytosis^59,60^. However, enrichment of SIGN-R1, C1q, C4, and C3 in splenic MZ SIGN-R1^+^ LRs could dramatically enhance the simultaneous recognition of pneumococcal CPSs with SIGN-R1 and C1q, thus overcoming impairment of the initial pneumococcal recognition^61^.

The human spleen and the classical pathway are integral for protection against infection by *S. pneumoniae*^39,62–65^. In particular, the DC-SIGN-C1q complex could also mediate a classical complement pathway against *S. pneumoniae* in the spleen, representing a potential protection mechanism in the human spleen^24^. Indeed, the replaced DC-SIGN on splenic MZs of DC-SIGN^BMT^/SIGN-R1^TKO^ mice completely rescued the pneumococcal capture, the sequential activation and deposition of C3 against *S. pneumoniae* in splenic MZs, which was likely to be related to the DC-SIGN-induced complex of mouse C1q and C4. Previous studies have shown that recruitment of DC-SIGN into LRs with binding to viral glycoproteins or the gC1qR^29,31,66^ strengthens the possible formation of a tetramolecular complex of glycoproteins/DC-SIGN/C1q/gC1qR and thus increases binding capacity through the complex^24,67^. Therefore, similar to SIGN-R1, splenic MZ DC-SIGN^+^ LRs may also provide a favorable microenvironment for the DC-SIGN-mediated classical complement pathway by forming another tetramolecular complex of DC-SIGN, C1q, gC1qR, and C4, finally enhancing sequential C3 activation.

Because DC-SIGN is exclusively expressed on human splenic perifollicular zone macrophages^37^, the presence of DC-SIGN in cadaver splenic LRs indicates that DC-SIGN is expressed in LRs from human splenic perifollicular zone macrophages. Also, similar with the previous reports^32,68,69^, complements C1q, C4, and C3b were enriched in human splenic LRs. Therefore, these cadaver splenic perifollicular zone DC-SIGN^+^ LRs may also be important for the DC-SIGN-mediated classical complement system against *S. pneumoniae*, explaining why the spleen and the classical pathway are integral for protection against infections to *S. pneumoniae* in humans^63–65^.

The importance of LRs has been restricted to their roles in receptor-mediated intracellular events from the plasma membrane, so far. However, splenic MZ LRs provide bidirectional platforms not only for usual events associated with the intracellular milieu, for example, recognition and phagocytosis of pathogens in vivo, but also unusual events associated with the extracellular milieu, such as the complement system, orchestrating early and complicated host responses to microbial infection. Thus, the spleen could be equipped with the most sensitive system to target various pathogens, such as *S. pneumoniae*^35,36^ and human immunodeficiency virus-1^37,38^. These findings can explain how such a small portion of splenic SIGN-R1^+^ macrophages (<0.05%) helps the spleen to efficiently protect hosts against *S. pneumoniae*, providing insights into the involvement of LRs in the innate immune system and the roles of DC-SIGN in the human spleen.

## Materials and methods

### Bacterial strains

*Streptococcus pneumoniae* capsular serotype 14 (DCC1490), the unencapsulated *S. pneumoniae* Tn*916* mutant Spn14.H, and *Staphylococcus aureus* strain (RN4220) were used, all of which were kindly provided by Professor Vincent A. Fischetti of Rockefeller University (New York, USA).

### Cell culture

DCEK, a mouse L-cell fibroblast line, and human embryonic kidney (HEK293T) cells were cultured in RPMI-1640 medium, and CHO cells were cultured in Dulbecco’s modified Eagle’s medium, supplemented with 10% fetal bovine serum, 100 U/mL penicillin G, and 100 mg/mL streptomycin. Stable DCEK transfectants expressing complementary (cDNA) for DC-SIGN or SIGN-R1 (DCEK_DC-SIGNS and DCEK_SIGN-R1 in the figures, respectively) and stable CHO transfectants expressing cDNA for Neo, or SIGN-R1 (CHO_Neo, or CHO_SIGN-R1 in the figures, respectively) were used.

### In vivo animal studies

Female C57BL/6 mice (6–10 weeks old, weighing 16–20 g) were purchased (The Jackson Laboratory, Bar Harbor, ME, USA) and housed under specific pathogen-free conditions. SIGN-R1 (CD209b)-KO mice were kindly provided by the Consortium for Functional Glycomics (http://www.functionalglycomics.org). DC-SIGN transgenic donor mice were provided by the Rockefeller Gene Targeting Resource Center and identified by PCR using DC-SIGN gene primers forward (5′-CgggATCCgAgTggggTgACATgAgTgACT-3′) and reverse (5′-ACgCgTCgACAAAAgggggTgAAgTTCTgCTACg-3′). For in vivo experiments using animal models, all studies were approved by the Institutional Animal Care and Use Committee of Konkuk University (permit number: KU11107) and performed in strict accordance with the approved guidelines for animal care and animal experimentation. Animal welfare was overseen by local committees. Mice were housed in a temperature-controlled room with an automated darkness–light cycle system and had ad libitum access to food and water. Mice were challenged with 1 × 10^8^ colony-forming unit of *S. pneumoniae* suspended in 100 mL of PBS in PBS intravenously. Prior to tissue dissection, the mice were sacrificed via euthanasia using an overdose of CO_2_ with a flow rate 20% of the cage volume per minute, following AVMA Guidelines for the Euthanasia of Animals.

### Bacterial growth conditions and fluorescent labeling

*Streptococcus pneumoniae* capsular serotype 14 (DCC1490), the unencapsulated *S. pneumoniae* Tn*916* mutant Spn14.H (mt-*Pn14*), and *Staphylococcus aureus* were grown in brain heart infusion broth (DIFCO) to mid-logarithmic phase (18 h, 37 °C). Bacteria were inactivated with 50 μg/mL mitomycin C (Sigma) for 1 h and suspensions of 10^9^ bacteria were labeled with PKH26 following the manufacturer’s instructions or with 5 mM CFSE (Sigma-Aldrich) for 30 min at 37 °C. The fluorescent bacteria were injected intravenously into mice or incubated with cells.

### Cellular and tissue immunofluorescence microscopy

Cells on coverslips or 10 μm OCT (Tissue-Tek) frozen spleen sections were fixed with 100% acetone (10 min, room temperature) and stained, where indicated, with DAPI (4′,6-diamidino-2-phenylindole) (blue) or FITC-, PE-, AMCA-, or Alexa Fluor-conjugated donkey anti-chicken IgY, goat anti-hamster IgG, donkey anti-rabbit IgG, goat anti-rat IgG, and streptavidin as secondary reagents (purchased from Abcam, Jackson ImmunoResearch Laboratories, or Molecular Probes). Cells and spleen sections were examined for fluorescence with a deconvolution fluorescence BX61-32FDIC microscope (Olympus Corp., Tokyo, Japan). Images were acquired with a Coolsnap^*EZ*^ system (Roper Scientific, Inc., AZ, USA).

### Flow cytometry

Cells used in FACS analysis were detached with 1 mM EDTA in PBS for 10 min and pre-incubated 10 min with 2.4G2 monoclonal antibody at 4 °C to block Fc receptors. Cells or *S. pneumoniae* were incubated with 3–5% mouse or human serum in 200 μL TC buffer (10 mM Tris-HCl, 140 mM NaCl, 2 mM CaCl_2_, 2 mM MgCl_2_, and 1% bovine serum albumin [BSA]), and complement binding to cells or organisms was detected by immunostaining with respective antibodies for 30 min at 4 °C. Cytometric analysis was performed using a FACScan (Becton Dickinson, San Jose, CA, USA) and CellQuestPro (BD Biosciences). Subsequent data analysis was performed with CellQuestPro (BD Biosciences).

### *Streptococcus pneumoniae* uptake and decomposition analysis in vitro and in vivo

Cells (1 × 10^5^) were incubated with mitomycin C-treated and CFSE-labeled *S. pneumoniae* (1 × 10^6^) for the indicated times at 37 °C, and bacterial binding to cells and cytoplasmic decomposition of capsular polysaccharides of *S. pneumoniae* were examined with a fluorescence microscope. Mice were intravenously injected with mitomycin C-treated and CFSE-labeled *S. pneumoniae* (1 × 10^8^) for the indicated times^39,41^, and bacterial binding and decomposition were examined on SIGN-R1^−^ or SIGN-R1^+^ cells of spleen tissues.

### Quantification of bacterial decomposition ratios

To quantify the pneumococcal decomposition ratio, the number of pneumococcal-bound or -decomposing cells was counted, and the average percentage of pneumococcal-decomposing cells from the total number of pneumococcal-bound cells was calculated in five areas for each sample from five independent experiments.

### Cell fractionation and purification of LRs with sucrose gradients

Tissues from mice or cells were solubilized in 2 mL of 1% Triton X-100 (Junsei Chemical Co., Ltd.) in MES-buffered saline (MBS, 25 mM MES, pH 6.5, 150 mM NaCl). After homogenizing with 10 up-and-down strokes of a tight-fitting Dounce homogenizer, the tissue or cellular extracts were adjusted to 4 mL with sucrose concentrations of 45% or 40%, respectively, and overlaid with 4 mL of 30% sucrose and 4 mL of 5% sucrose in MBS. The sucrose gradient was formed by centrifugation at 200,000 × g for 18–20 h at 4 °C using a Beckman SW41ti rotor. After centrifugation, the sucrose gradients were fractionated into 12 fractions without pelleting, and an opaque buoyant band corresponding to the LRs was collected at the interface between the 30% and 5% sucrose gradients. The same quantity of proteins from each fraction was used for immunoblotting analysis.

### Plasmids and transfection

Clone pMX DC-SIGN was a gift from Dr. Chae Gyu Park (Rockefeller University). This clone c DC-SIGN expression was monitored by immunoblotting analysis using anti-DC-SIGN antibodies. DCEK transfectants were transiently transfected with plasmids encoding dynamin II K44A (dominant-negative dynamin with a point mutation in the nucleotide-binding site, a gift from Professor Seung-Jae Lee, Seoul National University College of Medicine, Seoul, Korea) or with an empty pcDNA3 vector for 48 h, using Lipofectamine Plus reagent (Invitrogen Life Technologies, Carlsbad, CA, USA) according to the manufacturer’s specifications.

### SIGN-R1 TKO mouse generation

SIGN-R1 TKO or isotype control mice were generated by intravenous injection of 100 μg 22D1 antibody or isotype hamster IgG for 48 h. The 22D1 antibody selectively and transiently depleted the surface SIGN-R1 molecule, but not SIGN-R1^+^ macrophages in the splenic MZ, permitting analysis of its function in vivo, as in a previous report^70^.

### DC-SIGN transgenic mouse generation

DC-SIGN transgenic (DC-SIGN^BMT^/WT) mice were generated from the reconstitution of lethally irradiated C57BL/6 mice with bone marrow cells of DC-SIGN transgenic donor mice. SIGN-R1-depleted DC-SIGN transgenic (DC-SIGN^BMT^/SIGN-R1^TKO^) mice were generated from the intravenous injection of 22D1 for 48 h into DC-SIGN^BMT^/WT mice.

### Complement C3-depleted mice

Control mice were obtained by intraperitoneally injecting 60 U/kg of cobra venom factor one day prior to the experimental challenge. C3 depletion was confirmed by Western blot analysis for C3 by using sera.

### Mouse infection studies

Mitomycin C-treated fluorescent bacteria (1 × 10^8^) were intravenously administered to mice for the indicated times. Mice were sacrificed, and spleen sections were examined by deconvolution fluorescence microscopy.

### Assay for in vivo and in vitro C3 processing

To quantify C3 processing in tissues in vivo, 1 × 10^8^ *S. pneumoniae* were injected intravenously, and tissue lysates were collected at the indicated times, separated by sodium dodecyl sulfate polyacrylamide gel electrophoresis (SDS-PAGE), and immunoblotted with polyclonal anti-mouse C3 antibodies. For in vitro C3 processing, 1 × 10^8^ *S. pneumoniae* were incubated with 5% NHS in TC buffer (140 mM NaCl, 2 mM CaCl_2_, 2 mM MgCl_2_, 10 mM Tris, pH 7.5, supplemented with 1% BSA) for 30 min at 37 °C. The bacteria were washed, mixed, and boiled with 20 µL of 2× SDS sample buffer. Bacterial lysates were separated by SDS-PAGE and immunoblotted with polyclonal anti-mouse C3 antibodies. These antibodies detected the components of native C3, αC3, and βC3, as well as the fragments of αC3 (molecular weights in humans: 113 kDa for αC3, 104 kDa of αC3′, 63 kDa and 41 kDa for iC3b fragments, and 75 kDa for βC3; molecular weights in rats: 120 kDa for αC3, 70 kDa and 43 kDa for iC3b fragments, and 65 kDa for βC3) that are generated during C3 processing by C3 convertases by immunoblotting analysis. In the steady state, αC3 and βC3 are predominant, with a few larger iC3b. With activation, αC3 and larger iC3b are rapidly processed into smaller fragments, including 43 kDa iC3b, but not βC3, which serves as a loading control for the immunoblotting analysis, resulting in loss of most of the detectable αC3 as well as larger iC3b, but accumulation of the smaller iC3b in C3 activation in immunoblotting analysis^70^.

### Western blot analysis and immunodetection

Tissues, cells, and bacteria were lysed in RIPA buffer (150 mM NaCl, 50 mM Tris-HCl, pH 8.0/1% Nonidet P-40/0.5% sodium deoxycholate/0.1% SDS) supplemented with 0.2% protease inhibitor cocktail (Sigma), and lysed samples were mixed with an equal volume of 2× SDS sample buffer containing 2-mercaptoethanol and boiled at 95 °C for 5 min (100 °C for 10 min). Diluted sera (1:50) or fractions of LRs were mixed with 2× or 5× SDS sample buffer with 2-mercaptoethanol and boiled at 95 °C for 10 min (100 °C for 10 min). The samples were separated by SDS-PAGE on 4–15% gradient gels and transferred to polyvinylidene difluoride membranes, followed by incubation with antibodies. Antibody-reactive bands on the blots were visualized with peroxidase-labeled secondary antibodies, followed by treatment with West-ZOL plus (Intron) or Immobilon (Millipore).

### Human cadaver spleen studies

Human cadaveric spleens were donated from the Department of Anatomy, School of Medicine, Konkuk University (Seoul, Korea).

### Software used in this study

Fluorescent images were analyzed with the MetaMorph software (Universal Imaging). Immunoblotting signals were detected using LAS-4200 (Fuji Film).

### Statistical analyses

The number of cells binding to bacteria and the number of cells decomposing bacteria were counted. The average percentage of pneumococcal decomposition of the total pneumococcal binding to cells was calculated in five areas of each sample from three independent experiments and shown in the indicated graphs. Data are presented as mean ± SD. Statistical significance between groups were determined by two-way analysis of variance, followed by Tukey’s post hoc tests and unpaired Student’s *t* tests with a two-tailed test. *p* Value < 0.05 was taken to indicate statistical significance (not significant, ns; *p < 0.05; **p < 0.01; ***p < 0.001)

## Supplementary information

author-contribution-form

supplementary Figure 1

supplementary Figure 2

supplementary Figure 3

supplementary Figure 4

supplementary Figure 5

supplementary Figure 6

supplementary Figure 7

Supplemental Material File #1
