# Supplementary material for: Dominant role of splenic marginal zone lipid rafts in the classical complement pathway against S. pneumoniae
Source: Cell Death Discov. 2019 Sep 9;5:133. doi: 10.1038/s41420-019-0213-3 (PMC6733876; doi:10.1038/s41420-019-0213-3)
Supplement: Supplementary file 1 — author-contribution-form [file 41420_2019_213_MOESM1_ESM.pdf]

DECLARATION OF CONTRIBUTIONS TO ARTICLE

ADMC

Manuscript Number:	Journal Name:	
CDDISCOVERY-00675	*Cell Death Discovery*	(the 'Journal')

Proposed Title of the Contribution:

Dominant Role of Splenic Marginal Zone Lipid Rafts in the Classical Complement Pathway against S. pneumoniae (the 'Contribution)

Author(s):

Seung Woo Yang, Jin-Yeon Park, Hyeongjwa Choi, Tae Jin Yun, Woo-Sung Choi, Min-Kyung Kim, Yun Kyung Lee, Min Park, JIN YIHWA, Jin Soo Joo, In-Soo Choi, Seung Hwa Park, Han Sung Hwang, Young-Sun Kang (the 'Authors')

For all *CDDis*covery articles, each person named as an author in the published version must be able to show he or she has contributed substantially to the article.

Authorship credit should be based on 1) substantial contributions to conception and design, acquisition of data, or analysis and interpretation of data; 2) drafting the article or revising it critically for important intellectual content; and 3) final approval of the version to be published. Authors should meet conditions 1, 2 and 3.

Any person who cannot be shown to have made a substantial contribution to the article cannot be listed as an author in the final version. The name of any person who is deemed to have made a minor contribution can, however, appear in the Acknowledgments section of the article.

Please complete the table below to indicate the contributions of all named authors to the manuscript.

Author Full Name:	Specification of Contribution to the Manuscript:
SEUNG WOO YANG	acquisition data, drafting the article, final approval
JIN-YEON PARK	acquision data, final approval
HYEONGJWA PARK	acquisition data, analysis data, final approval
TAE JIN YUN	acquisition data, analysis, final approval
WOO-SUNG CHOI	Interpretation of data, revising draft, final approval
MIN-KYUNG KIM	acquisiton data, final approval
YUN KYUNG LEE	acquisiton data, final approval
MIN PARK	acquisiton data, final approval
JIN YIHWA	acquisiton data, final approval
JIN SOO JOO	acquisiton data, final approval
IN-SOO CHOI	analysis and interpretation data, revising draft, final approval
SEUNG HWA PARK	analysis and interpretation data, revising draft, final approval
HAN SUNG HWANG	analysis and interpretation data, revising draft, final approval
YOUNG-SUN KANG	conception and design, analysis data. revising draft, final approval

Please complete the table below to indicate the contributions of all named authors to the figures.

Figure 1:

YSK, SWY, HC designed and assembled the figure; JYP, MKK, YKL, MP cultured DCEK_DC-SIGN transfectant cell line, performed immunofluorescence staining and in vitro analysis; SWY performed FACs; HC, TJY, WSC, JY, JSJ maintain DC-SIGNBMT/SIGN-R1TKO mice and performed in-vivo analysis; SWY, YKL, HC removed cadevar spleen and performed western blot; YSK, ISC, HSH, SHP revised figure

Figure 2:

YSK, SWY, HC designed and assembled the figure; JYP, MKK, YKL, MP cultured DCEK_SIGN-R1 transfectant cell line, performed immunofluorescence staining and in vitro analysis; SWY performed FACs; HC, TJY, WSC, JY, JSJ maintain wild type/SIGN_R1 K.O mice and performed in-vivo analysis; ISC, HSH, SHP revised figure.

Figure 3:

YSK, SWY, HC designed and assembled the figure; JYP, MKK, YKL, MP cultured DCEK_SIGN-R1 transfectant cell line, performed immunofluorescence staining and western blot after MitC-Pn14 or MβCD treatment; HC, TJY, WSC, JY, JSJ maintain mice and performed in-vivo analysis including immunofluorescence staing or western blot; ISC, HSH, SHP revised figure.

Figure 4:

YSK, SWY, HC designed and assembled the figure; HC, TJY, WSC, JY, JSJ maintain mice and performed in-vivo analysis of western blot; ISC, HSH, SHP revised figure.

Figure 5:

YSK, SWY, HC designed and assembled the figure; JYP, MKK, YKL, MP cultured DCEK_WT or _SIGN-R1 transfectant cell line, and performed immunofluorescence staining; SWY performed FACs; HC, TJY, WSC, JY, JSJ maintain mice and performed in-vivo analysis of complement western blot; ISC, HSH, SHP revised figure.

Figure 6:

YSK, SWY, HC designed and assembled the figure; JYP, MKK, YKL, MP cultured DCEK_WT or _SIGN-R1 transfectant cell line, CHO- transfectant cell line and performed immunofluorescence staining; SWY performed FACs; HC, TJY, WSC, JY, JSJ maintain mice and performed in-vivo analysis of spleen immunofluorescence and western blot; ISC, HSH, SHP revised figure.

Please complete the table below to indicate the contributions of all named authors to the figures.

Figure 7:

YSK, SWY, HC designed and assembled the figure; JYP, MKK, YKL, MP cultured DCEK_WT or _DC-SIGN transfectant cell line, HEK 293T line and performed immunofluorescence staining and western blot; SWY performed FACs; HC, TJY, WSC, JY, JSJ maintain SIGN-R1 KO, SIGN-R1TKO, and DC-SIGNBMT/SIGN-R1TKO mice and performed in-vivo analysis of spleen immunofluorescence and western blot; SWY, YKL, HC removed cadevar spleen and performed western blot; ISC, HSH, SHP revised figure.

Signed for and on behalf of the Author(s):	Print Name:	Date:
Kang Young Sun	YOUNG-SUN KANG	2019.04.13
