# Supplementary figures and images for: Dominant role of splenic marginal zone lipid rafts in the classical complement pathway against S. pneumoniae
Source: Cell Death Discov. 2019 Sep 9;5:133. doi: 10.1038/s41420-019-0213-3 (PMC6733876; doi:10.1038/s41420-019-0213-3)

Figure S1

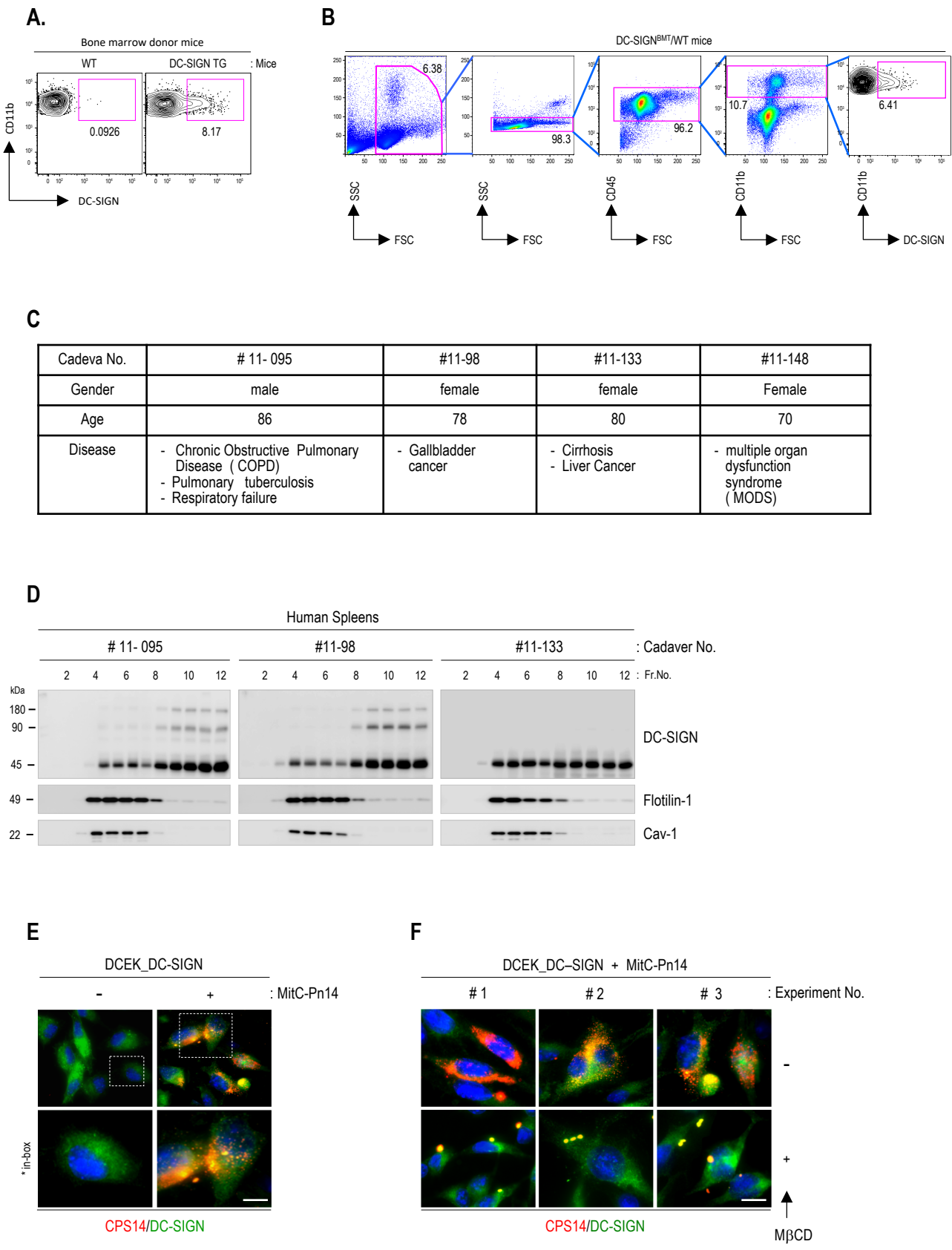

Supplement: Supplementary file 2 — supplementary Figure 1 [file 41420_2019_213_MOESM2_ESM.pdf]

Figure S2

A

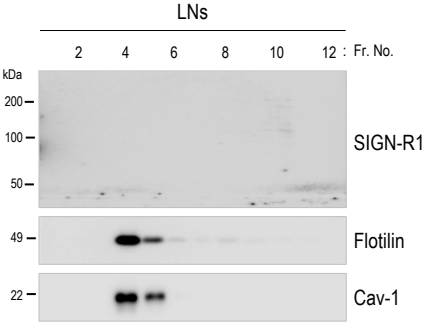

B

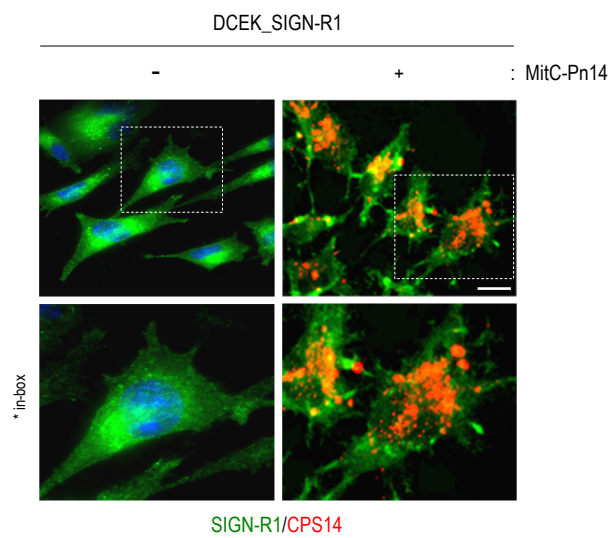

Supplement: Supplementary file 3 — supplementary Figure 2 [file 41420_2019_213_MOESM3_ESM.pdf]

Figure S3

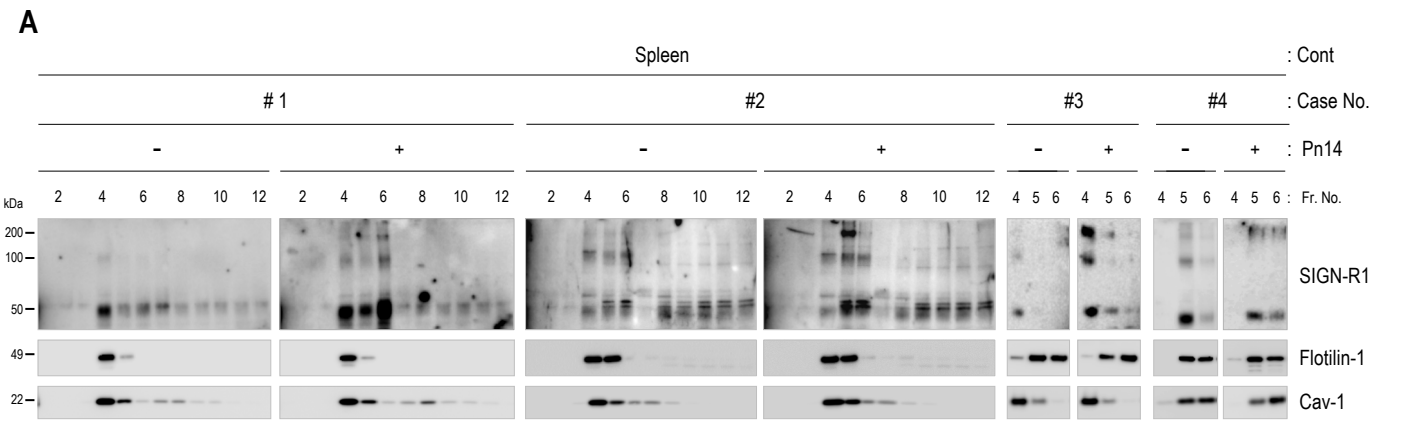

Supplement: Supplementary file 4 — supplementary Figure 3 [file 41420_2019_213_MOESM4_ESM.pdf]

Figure S4

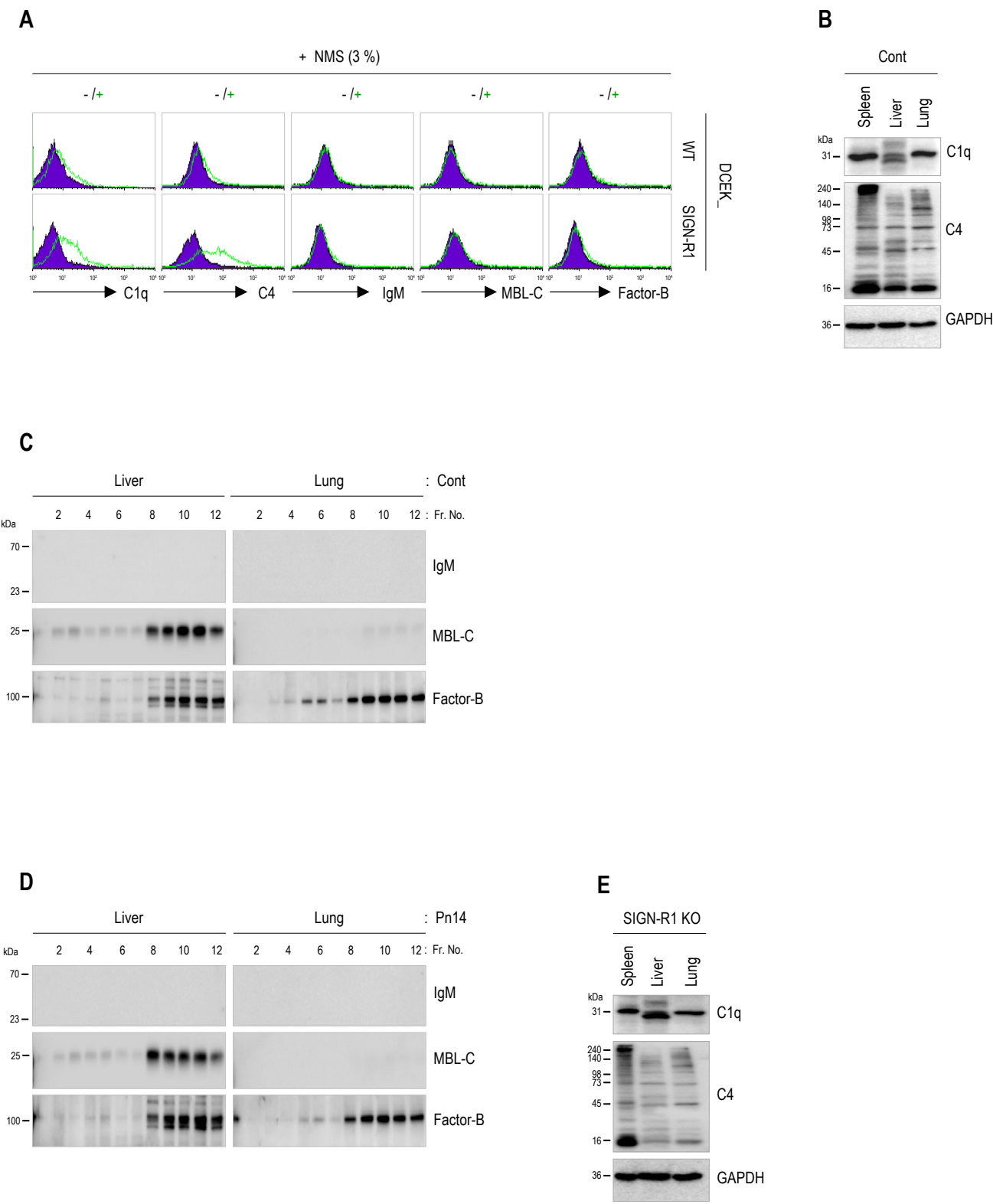

## Figure S4

# F

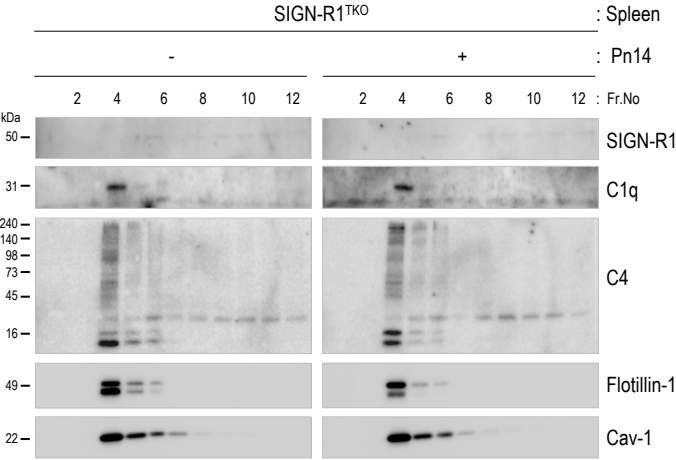

Supplement: Supplementary file 5 — supplementary Figure 4 [file 41420_2019_213_MOESM5_ESM.pdf]

Figure S5

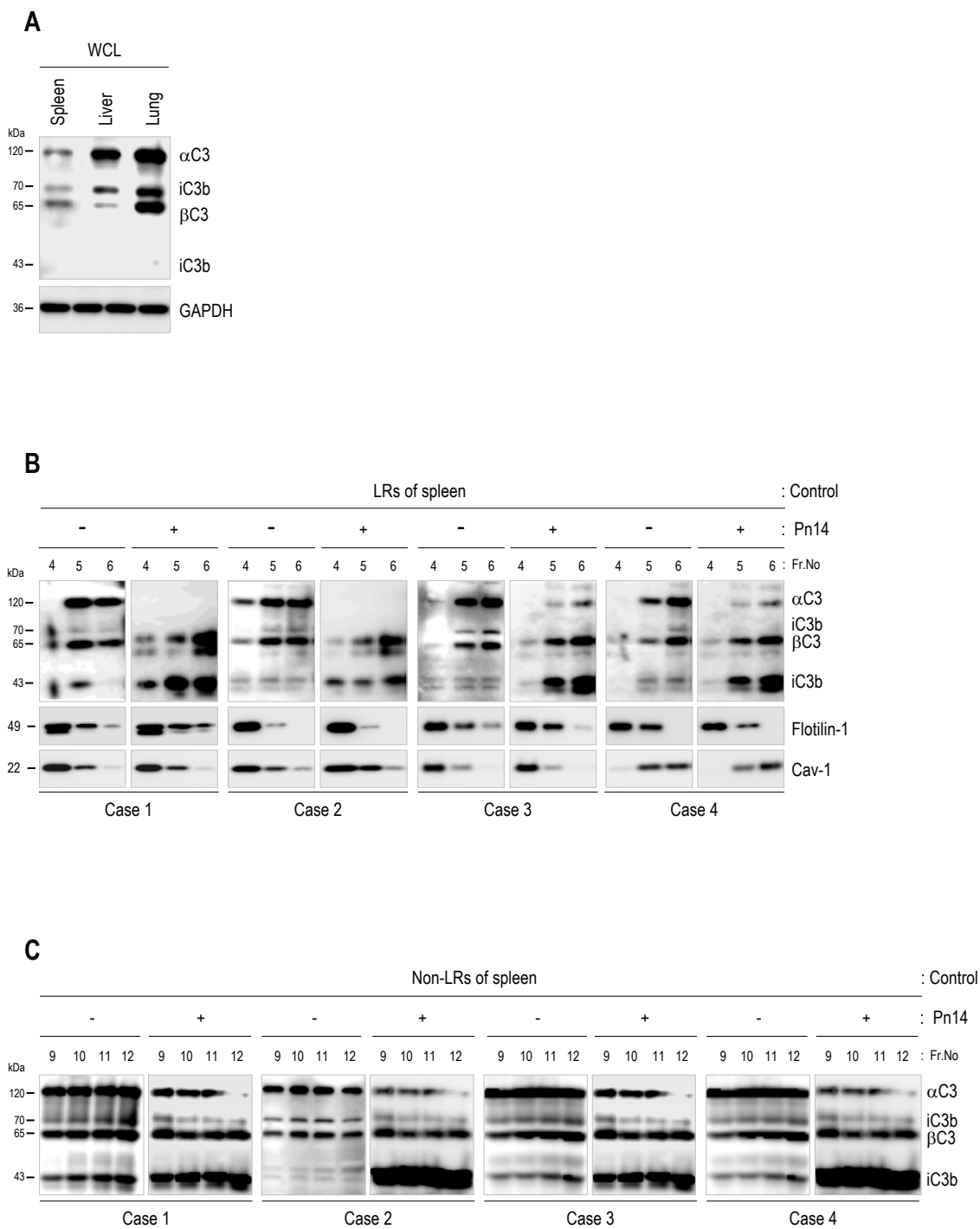

Figure S5

D

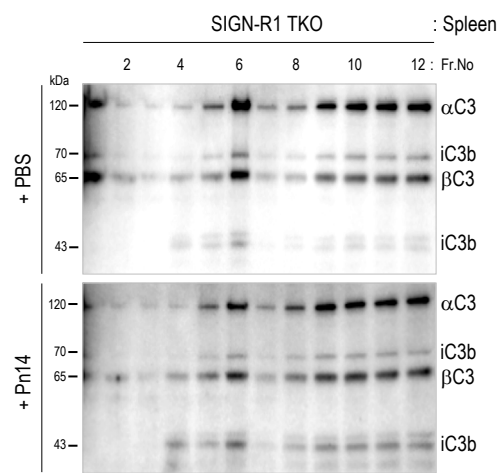

E

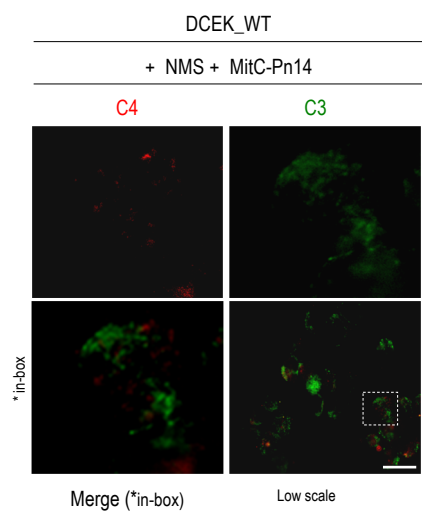

Supplement: Supplementary file 6 — supplementary Figure 5 [file 41420_2019_213_MOESM6_ESM.pdf]

# Figure S6

A

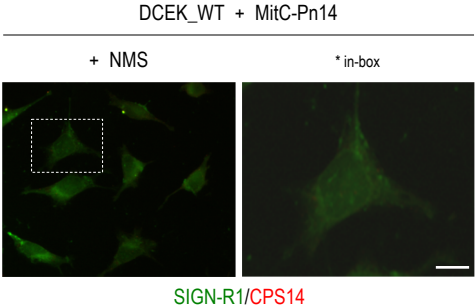

B

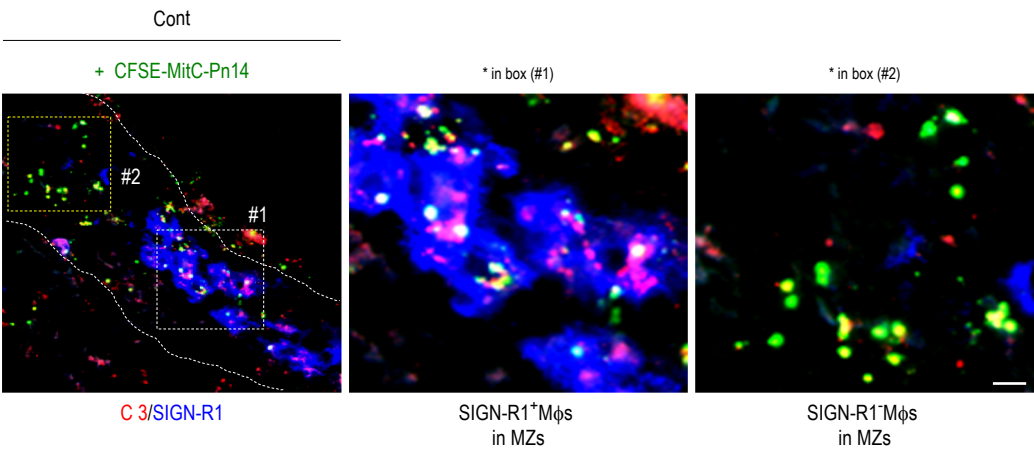

C

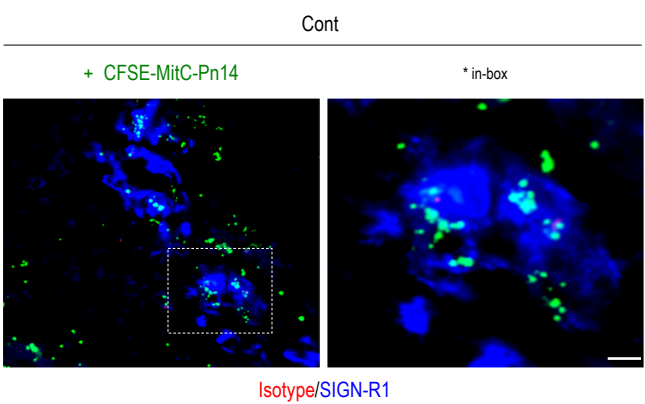

Supplement: Supplementary file 7 — supplementary Figure 6 [file 41420_2019_213_MOESM7_ESM.pdf]

Figure S7

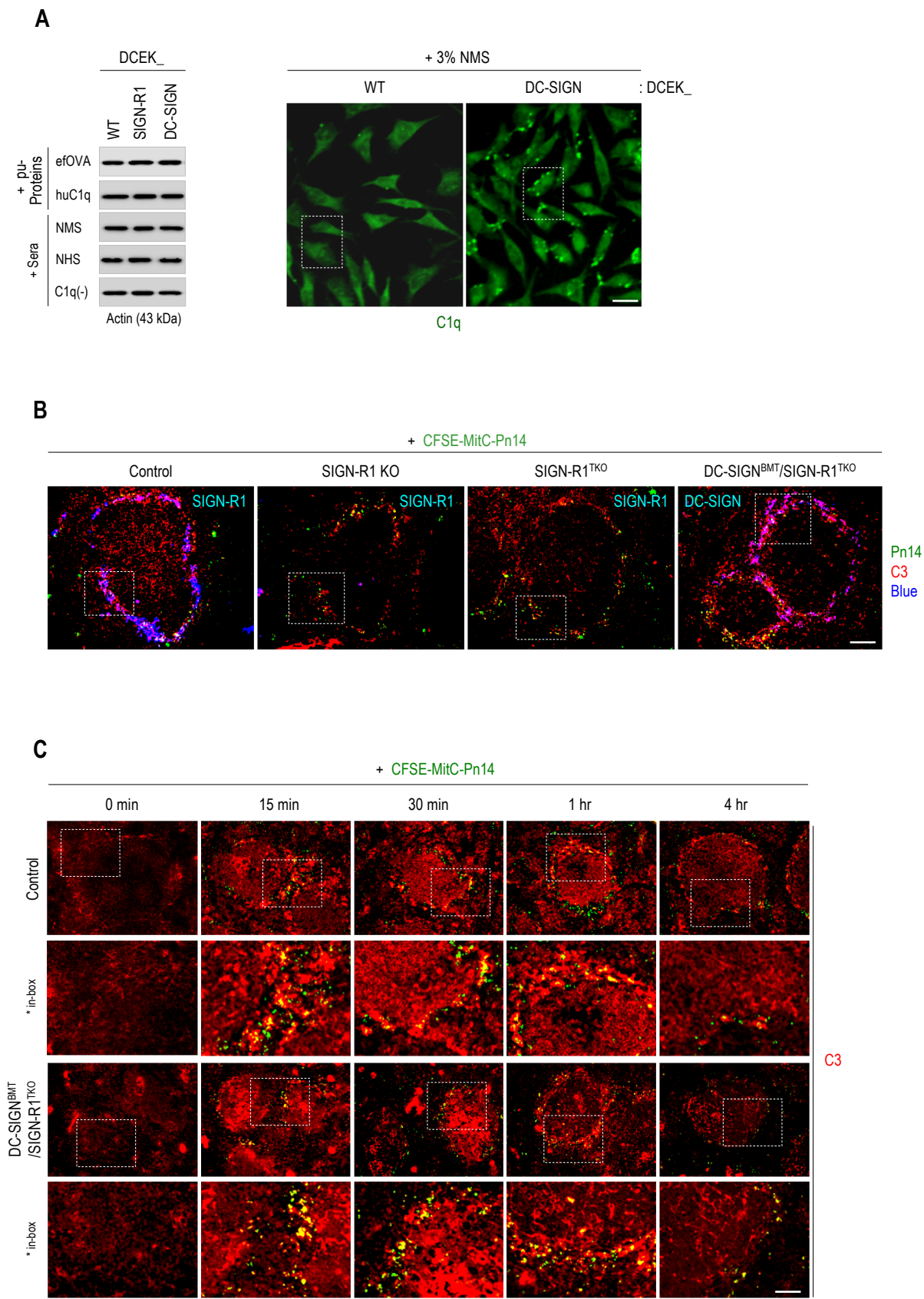

Figure S7

D

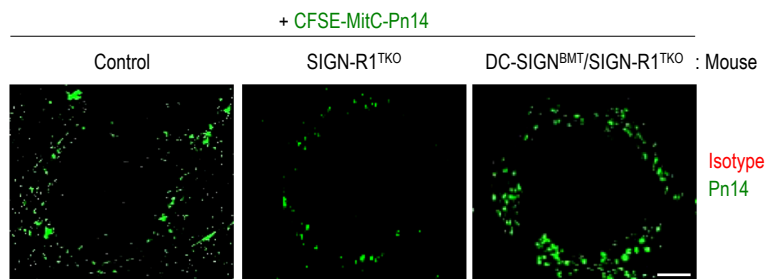

E

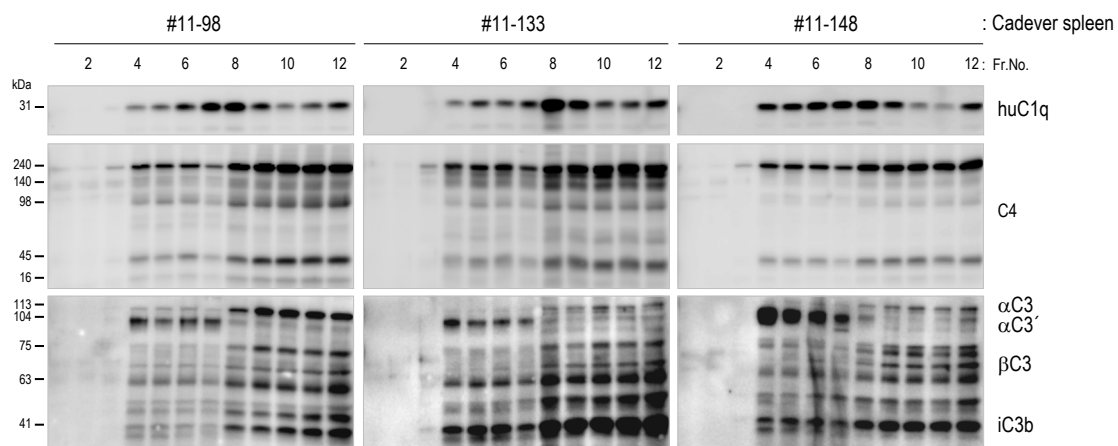

Supplement: Supplementary file 8 — supplementary Figure 7 [file 41420_2019_213_MOESM8_ESM.pdf]
