# Supplementary material for: Dominant role of splenic marginal zone lipid rafts in the classical complement pathway against S. pneumoniae
Source: Cell Death Discov. 2019 Sep 9;5:133. doi: 10.1038/s41420-019-0213-3 (PMC6733876; doi:10.1038/s41420-019-0213-3)
Supplement: Supplementary file 9 — Supplemental Material File #1 [file 41420_2019_213_MOESM9_ESM.docx]

SUPPLEMENTAL INFORMATION

**S1 Fig. Distribution of DC-SIGN in splenic lipid rafts and its role in the uptake and decomposition of *S. pneumoniae*.** (A) Representative gating strategy for peripheral blood mononuclear cells from wild-type or DC-SIGN transgenic mice. Cells were immunostained for CD45, CD11b, and DC-SIGN and assessed by FACS. (B) As in (A), but peripheral blood mononuclear cells were prepared from wild-type mice reconstituted with bone marrow cells from DC-SIGN transgenic mice. (C) The information for three cadavers is listed. (D) After sucrose gradient ultracentrifugation of splenic lysates from three cadavers, the fractionated samples (*from top to bottom*) were immunoblotted for DC-SIGN, flotilin-1, and caveolin-1. (E) Following 16 hr of serum starvation, DCEK_DC-SIGN transfectants were incubated with PBS or mitomycin C-treated *S. pneumoniae* type 14 (1 × 10^6^, 15 hr, 37°C), followed by immunostaining for DC-SIGN (green) and CPS14 (red). (F) As in (F-1), but DCEK_DC-SIGN transfectants were pretreated with PBS or MβCD (10 mM, 3 hr). Scale bars E,F, 20µm.

**S2 Fig. Distribution of SIGN-R1 in lipid rafts of lymph nodes from SIGN-R1 KO mouse and the uptake and decomposition of *S. pneumoniae* in SIGN-R1 transfectant.** (A) After sucrose gradient ultracentrifugation of lysates from lymph nodes of SIGN-R1 KO mice, the fractionated samples (*from top to bottom*) were immunoblotted for SIGN-R1, flotilin-1, and caveolin-1. (B) Following 16 hr of serum starvation, DCEK_SIGN-R1 transfectants were incubated with PBS or mitomycin C-treated *S. pneumoniae* type 14 (1 × 10^6^, 15 hr, 37°C), followed by immunostaining for SIGN-R1 (green) and CPS14 (red). Scale bars B, 20µm.

**S3 Fig. Accumulation and multimerization of SIGN-R1 in splenic lipid rafts following exposure to CPS14 from *S. pneumoniae in vivo*.** (A) Mice were injected intravenously with live *S. pneumoniae* (1 × 10^8^, 1 hr), and their spleens were fractionated by sucrose gradient ultracentrifugation. The fractionated samples (*from top to bottom*) were immunoblotted for SIGN-R1, flotilin-1, and caveolin-1. Results from cases 1 and 2 are presented for whole fractions, but results from cases 3 and 4 are presented for lipid raft fractions.

**S4 Fig. Predominant distribution of complement C1q and C4 in splenic lipid rafts and their upregulation after *S. pneumoniae* challenge in a SIGN-R1-dependent manner *in vivo*.** (A) DCEK_WT and _SIGN-R1 cells were detached with EDTA, incubated with 3% normal mouse serum for 30 min at 37°C and assessed by FACS for C1q, C4, IgM, MBL-C, and factor-B. (B) Lysates from the spleen, liver, and lungs of control mice were immunoblotted for C1q, C4, and GAPDH. (C) Liver and lung samples from control mice were fractionated by sucrose gradient ultracentrifugation, and the fractionated samples (*from top to bottom*) were immunoblotted for IgM, MBL-C, and factor-B. (D) As in (C), but mice were injected intravenously with live *S. pneumoniae* (Pn14; 1 × 10^8^, 1 hr). (E) As in (B), but tissue lysates from SIGN-R1 KO mice were used. (F) As in (C), but spleens of isotype control IgG or 22D1-treated mice (100 μg, intravenous *injection*, 24 hr) were fractionated and immunoblotted for SIGN-R1, C1q, C4, flotilin-1, and caveolin-1. (G) As in (F), 22D1-treated mice were injected intravenously with PBS or Pn14 (1 × 10^8^, 1 hr).

**S5 Fig. Dominant C3 activation in splenic lipid rafts in a SIGN-R1-dependent manner following *S. pneumoniae* challenge *in vivo* and *in vitro*.** (A) Lysates from tissues of control mice were immunoblotted for C3 and GAPDH. (B) After mice were injected intravenously with PBS or live *S. pneumoniae* (Pn14; 1 × 10^8^, 1 hr), their spleens were fractionated by sucrose gradient ultracentrifugation and the splenic lipid raft fractions (fractions 4–6) were immunoblotted for C3, flotilin-1, and caveolin-1. (C) As in B, but splenic non-lipid raft fractions (fractions 9–12) were used. (D) Sera of wild-type and SIGN-R1 KO mice were immunoblotted for C3. (E) As in (A), but SIGN-R1 KO mice were used. (F) Spleens from isotype control IgG or 22D1-treated mice were fractionated by sucrose gradient ultracentrifugation, and the fractionated samples (*from top to bottom*) were immunoblotted for C3. (G) As in (F), but mice were injected with PBS or Pn14 (1 × 10^8^, 1 hr). (F) DCEK_WT cells were incubated with mitomycin C-treated *S. pneumoniae* type 14 (1 × 10^7^, 1 hr) with 5% normal mouse serum in TC buffer for 15 min at 37°C. C4 (red) and C3 (green) binding were assessed by microscopy after fixing cells with 1% paraformaldehyde. A representative cell is shown in the inset. Scale bars E, 20µm.

**S6 Fig. SIGN-R1-mediated C3 activation and opsonization of *S. pneumoniae* in the uptake and decomposition of the bacteria by SIGN-R1^+^ Cells *in vitro* and *in vivo*.**

(A) DCEK_WT cells were incubated with mitomycin C-treated *S. pneumoniae* type 14 (MitC-Pn14; 1 × 10^6^, 15 hr, 37°C) in the presence of normal mouse serum, and cells were immunostained for SIGN-R1 (green) or CPS14 (red), followed by microscopic analysis. (B) Carboxyfluorescein succinimidyl ester (CFSE)-labeled MitC-Pn14 (1 × 10^8^; green) were injected intravenously into control mice for 1 hr, and spleen sections from these mice were immunostained for SIGN-R1 (blue) or CPS14 (red). Representative areas of SIGN-R1^+^ or SIGN-R^-^ macrophages in the splenic MZ are highlighted in the insets (*inset #1 or #2, respectively). (C) As in F-1, but spleen sections were immunostained for SIGN-R1 (blue) with isotype control IgG of anti-CPS14 antibodies (red). A representative area in the splenic MZs is highlighted in the inset. Scale bars A, 20µm; B, C, 40µm.

**Figure S7. The importance of splenic lipid rafts in a possible DC-SIGN-mediated classical complement pathway in response to *S. pneumoniae* challenge by interacting with C1q and C4.**

(A) (Left) Wild-type or DCEK transfectants were incubated with 5 μg endotoxin-free ovalbumin (efOVA), human C1q, or 3% serum (normal mouse serum, normal human serum, or C1q-depleted human serum [C1q(-) huSer]) in TC buffer for 1 hr at 37°C, and their lysates were immunoblotted for β-actin. (Right) Wild-type or DCEK_DC-SIGN transfectants were incubated with 3% NMS for 1 hr at 37°C and immunostained for C1q, followed by microscopic analysis. Representative areas are highlighted in Figure 7E (right). (B) Mitomycin C-treated and CFSE-labeled *S. pneumoniae* (CFSE-MitC-Pn14; 1 × 10^8^; green) were injected intravenously into control, SIGN-R1 KO, SIGN-R1^TKO^, or DC-SIGN^BMT^/SIGN-R1^TKO^ mice for 15 min, and their splenic sections were immunostained for C3 (red) and SIGN-R1 (green) or C3 (red) and DC-SIGN (green). The respective insets are presented in Figure 7J. (C) As in (B), but control or DC-SIGN^BMT^/SIGN-R1^TKO^ mice were injected intravenously for 0, 15, 30, 60, or 240 min and splenic sections were immunostained for C3 (red). Representative areas are highlighted in the insets. (D) As in (B), but splenic sections were immunostained with isotype control IgG of C3. (E) Spleens of three additional cadavers were fractionated by sucrose gradient ultracentrifugation, and the fractionated samples (*from top to bottom*) were immunoblotted for C1q, C4, C3, flotilin-1, and caveolin-1. Scale bars A, 20µm; B, C, D, 200µm.
